# Mechano‐Bactericidal Surfaces Achieved by Epitaxial Growth of Metal–Organic Frameworks

**DOI:** 10.1002/advs.202505976

**Published:** 2025-08-30

**Authors:** Zhejian Cao, Santosh Pandit, Francoise M. Amombo Noa, Jian Zhang, Wengeng Gao, Shadi Rahimi, Lars Öhrström, Ivan Mijakovic

**Affiliations:** ^1^ Department of Life Sciences Chalmers University of Technology Gothenburg SE‐41296 Sweden; ^2^ Wallenberg Initiative Materials Science for Sustainability Department of Life Sciences Chalmers University of Technology Gothenburg SE‐41296 Sweden; ^3^ School of Medicine Huanghe Science & Technology University Zhengzhou 450063 China; ^4^ Department of Chemistry and Chemical Engineering Chalmers University of Technology Gothenburg SE‐41296 Sweden; ^5^ Ecole des Sciences de la Santé Université Catholique D'Afrique Centrale Yaoundé B.P.1110 Cameroun; ^6^ The Novo Nordisk Foundation Center for Biosustainability Technical University of Denmark Kogens Lyngby DK‐2800 Denmark

**Keywords:** antibacterials, biofilm, mechano‐bactericidal surface, metal–organic framework, MOF‐on‐MOF

## Abstract

Mechano‐bactericidal (MB) surfaces have been proposed as an emerging strategy for preventing biofilm formation. Unlike antibiotics and metal ions that chemically interfere with cellular processes, MB nanostructures cause physical damage to the bacteria. The antibacterial performance of artificial MB surfaces relies on rational control of surface features, which is difficult to achieve for large surfaces in real‐life applications. Herein, a facile and scalable method is reported for fabricating MB surfaces based on metal–organic frameworks (MOFs) using epitaxial MOF‐on‐MOF hybrids as building blocks with nanopillars of less than 5 nm tip diameter, 200 nm base diameter, and 300 nm length. Two methods of MOF surface assembly, in situ growth and ex situ dropcasting, result in surfaces with nanopillars in different orientations, both presenting MB actions (bactericidal efficiency of 83% for *E. coli*). Distinct MB mechanisms, including stretching, impaling, and mechanical injury, are discussed with the observed bacterial morphology on the obtained MOF surfaces.

## Graphical abstract

Mechano‐bactericidal surfaces based on metal–organic frameworks (MOFs) are designed using a MOF‐on‐MOF strategy. Two methods of MOF surface assembly, in situ growth and ex situ dropcasting, are applied. Distinct mechano‐bactericidal actions are discussed, including stretching, impaling, and mechanical injury.

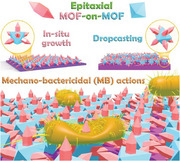

## 1 Introduction

Bacterial biofilms are the linchpin of persistent infections and biofouling.^[^ ^1^, ^2^ ^]^ In particular, medical indwelling devices, dental devices, and prostheses provide accessible surfaces conducive to biofilm development, contributing to ≈80% of chronic and nosocomial infections.^[^ ^3^ ^]^ Mitigation strategies based on chemical interference with cellular processes have only limited success as biofilm cells possess much higher resistance to antibiotics and the human immune system compared to their planktonic counterparts^[^ ^3^ ^]^. Furthermore, misuse of antibiotics has accelerated the spread of antimicrobial resistance (AMR), which is currently considered one of the largest threats to global health.^[^ ^4^ ^]^ Biofilm formation starts with bacterial attachment on surfaces, after which bacteria proliferate and adhere to each other within a self‐produced extracellular polymeric matrix.^[^ ^5^ ^]^ Therefore, preventing the initial attachment of bacteria to a surface could be an effective mitigation strategy to slow down or even preclude the formation of mature biofilms.^[^ ^6^, ^7^ ^]^

Mechano‐bactericidal (MB) surfaces have emerged as a material‐centric solution for preventing biofilm formation.^[^ ^8^ ^]^ Unlike antibiotics or metal ions that attack bacteria chemically, MB actions involve nanostructures inducing rupture and death of bacterial cells through physicomechanical interactions.^[^ ^9^ ^]^ MB actions can be found in natural and artificial nanostructured surfaces (Section S1, Supporting Information), such as the cicada wings,^[^ ^10^ ^]^ gecko skin,^[^ ^11^ ^]^ black silicon,^[^ ^12^ ^]^ and vertical graphene.^[^ ^13^ ^]^ In addition to their intrinsic bactericidal efficacy, nanostructured surfaces are reported to promote the effectiveness of antibiotics, which can potentially reduce antibiotic dosage.^[^ ^14^ ^]^ Nevertheless, real‐life applications of artificial MB surfaces are limited by complex fabrication processes, as nanostructures are required to provide MB efficacy.^[^ ^15^, ^16^ ^]^ For instance, we have previously demonstrated that optimal bacterial killing was achieved with graphene nanosheets aligned perpendicular to the surface, not thicker than 10 atom layers, and spaced so that bacterial cells cannot fit between adjacent spikes.^[^ ^17^ ^]^ However, the fabrication of such surfaces is typically expensive, laborious, and requires sophisticated equipment.^[^ ^18^ ^]^ These critical challenges impede the scalability and integration of MB surfaces into practical applications. We propose that metal–organic frameworks (MOFs), with their access to various morphologies, including sharp‐point shape crystal growth, could be used to create MB surfaces using a facile and economical fabrication process with the scalability required for real‐life applications.

MOFs are emerging porous materials with crystalline structures, designable geometry, tailorable chemical composition, and mild synthesis conditions.^[^ ^19^, ^20^ ^]^ Despite being relatively novel materials, the ton‐scale production of many MOFs has been reported,^[^ ^21^, ^22^, ^23^ ^]^ and size and morphology control is routine in industrial crystallization.^[^ ^24^ ^]^ Furthermore, MOFs are currently developed, academically and in start‐up companies, for various bio‐applications, such as drug delivery, enzyme immobilization, and biosensors.^[^ ^25^, ^26^ ^]^ Therefore, MOFs could be a promising candidate as building blocks for scalable MB surfaces. In this study, we control the orientation of MOFs based on biocompatible metal ions to create spike‐like surfaces with MB properties. This is a different approach from many MOFs that have been reported as potential antibacterial agents due to their ability to release toxic metal ions (Section S2, Supporting Information), including silver (Ag), copper (Cu), zinc (Zn), and cobalt (Co) ions.^[^ ^27^, ^28^ ^]^ Here we report a facile process to fabricate MOF MB surfaces through epitaxial growth of MOF‐on‐MOF hybrids with sharp‐tip features, using two biocompatible MOFs, MIL‐88B(Fe) and UiO‐66(Zr).^[^ ^29^, ^30^, ^31^ ^]^ These MOF particles presented a caltrop‐like 3D structure and were assembled on surfaces through in situ growth and ex situ dropcasting methods. The obtained MOF surfaces presented MB effects toward both Gram‐positive and Gram‐negative bacteria. Our approach and results cast light on the obstacles of achieving MB surfaces with rational‐controlled geometric features in a facile manner and open a novel sight to apply MOFs for antibacterial applications.

## 2 Rational Control of Geometric Features in MOF‐on‐MOF Surfaces

**Figure 1:**
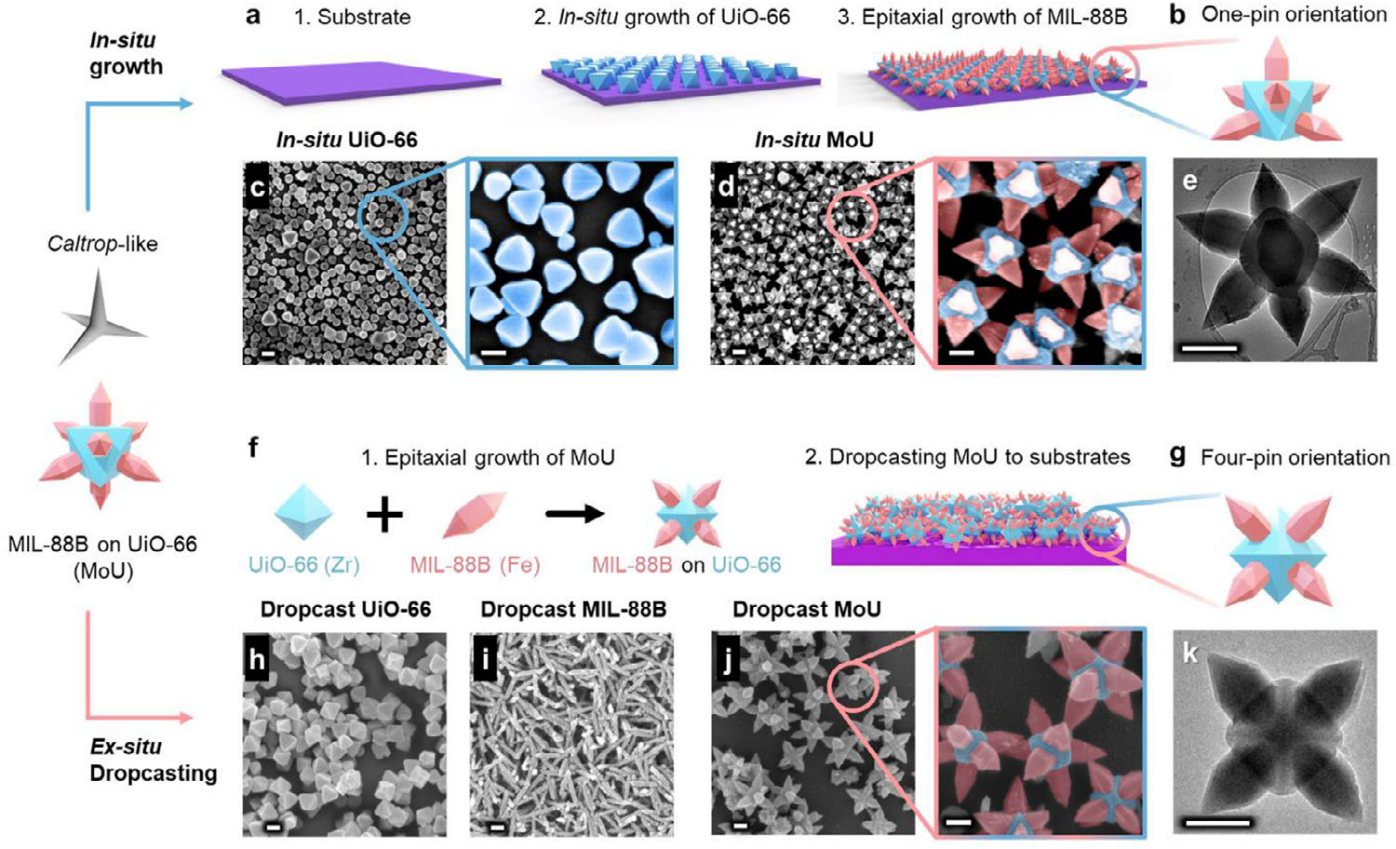
MOF mechano‐bactericidal (MB) surfaces. Caltrop‐like MIL‐88B‐on‐UiO‐66 (MoU) hybrids are used as the building blocks for MB surfaces through two assembly methods. a) Schematic showing MoU surfaces through in situ growth: 1. substrate, 2. in situ growth of UiO‐66 on the substrate, 3. epitaxial growth of MIL‐88B‐on‐UiO‐66 (MoU); b) Schematic illustration of the one‐pin up orientation in in situ MoU surfaces; SEM images of c) in situ UiO‐66 surface and d) in situ MoU surface after epitaxial growth of MIL‐88B; e) TEM image of MoU hybrid with one‐pin up orientation; f) Schematic showing MoU surfaces through ex situ dropcasting, 1. MoU hybrids through epitaxial growth, 2. Dropcasting MoU to the substrate; g) Schematic illustration of the four‐pin up orientation in dropcast MoU surfaces; SEM images of dropcast h) UiO‐66, i) MIL‐88B, and j) MoU, zoomed‐in SEM image false‐colored with UiO‐66 in blue and MIL‐88B in pink; k) TEM image of MoU hybrid with a four‐pin up orientation. Scale bar: 200 nm.

The size, orientation, and density of nanopatterns are critical for the performance of natural and artificial MB surfaces.^[^ ^8^, ^32^ ^]^ MIL‐88B(Fe) was selected as the nanopillar due to its nanoscale size, spike‐like geometry, and low cytotoxicity.^[^ ^29^ ^]^ However, arranging MIL‐88B nanopillars perpendicularly to be accessible to bacteria is challenging. Furthermore, MIL‐88B as nanopillars would have to be precisely spaced to avoid the bed‐of‐nails effect^[^ ^33^ ^]^ and intercalation of bacteria in between adjacent pillars.^[^ ^34^, ^35^ ^]^ Based on these criteria, we applied a core‐satellite MOF‐on‐MOF strategy, where UiO‐66 was used as the core and MIL‐88B as the satellite.^[^ ^36^, ^37^ ^]^ The caltrop‐like MIL‐88B‐on‐UiO‐66 (denoted as MoU) hybrids were assembled with two approaches, i.e., in situ growth and ex situ dropcasting, to achieve MOF MB surfaces shown in **Figure** **1**. The in situ growth approach (Figure 1a) resulted in a one‐pin up orientation (Figure 1b; Video S1, Supporting Information), where a UiO‐66 layer first in situ grew on the substrate, followed by the epitaxial growth of the MIL‐88B onto the UiO‐66 crystals. The length of the MIL‐88B nanopillars was ≈300 nm, and the base and tip diameters of the nanopillars were ≈200 nm and less than 5 nm, respectively, as shown in the transmission electron microscopy (TEM) image (Figure 1e). The pitch distance between the tips of vertical MIL‐88B nanopillars was ≈500 nm, which was controlled by the distance between the centers of UiO‐66 cores (Section S3, Supporting Information), as demonstrated in the scanning electron microscopy (SEM) images (Figure 1c,d). This position correlation between the nanopillars and the core MOF opens the possibility to rationally adjust the surface features in the MOF MB surfaces, as the distance between adjacent UiO‐66 particles and the length of the MIL‐88B can be modified (Section S4, Supporting Information).^[^ ^38^, ^39^, ^40^, ^41^ ^]^ The other method we used was dropcasting, which has been reported as one of the simplest ex situ methods for the fabrication of MOF thin films.^[^ ^42^ ^]^ In this approach, MoU hybrids were synthesized and then dropcast to substrates as illustrated in Figure 1f. When dropcast, due to the unique eight‐pin 3D structure of the MoU hybrid, the MoU tended to land with four nanopillars on the surface to achieve mechanical equilibrium, resulting in a four‐pin up orientation (Figure 1g; Video S2, Supporting Information), as demonstrated in the TEM image (Figure 1k). Notably, to achieve a fully covered surface, some areas of the dropcast MOF coating consisted of multilayered MoU, which could lead to a random orientation and distance of the MIL‐88B nanopillars (Figure 1j). Dropcast UiO‐66 (Figure 1h) and MiL‐88B (Figure 1i) surfaces lacked the critical geometric features of MB surfaces compared with MoU surfaces, including sharp nanostructures and vertical orientation. Both UiO‐66^[^ ^43^, ^44^ ^]^ and MIL‐88B^[^ ^45^ ^]^ are reported to be suitable for large‐scale production and are commercially available on the market.^[^ ^46^, ^47^, ^48^ ^]^ Since MoU synthesis is a combination of UiO‐66 and MIL‐88B production, the resulting material can be considered both scalable and economical. Furthermore, our MoU surface assembly methods required no sophisticated equipment and involved low fabrication temperatures, i.e., 120 °C for the MOF synthesis by in situ growth and room temperature (≈20 °C) by ex situ dropcasting. This allows for large‐scale MOF MB surfaces with a wide range of feasible substrates, especially materials that cannot stand high temperatures (Section S5, Supporting Information).

**Figure 2:**
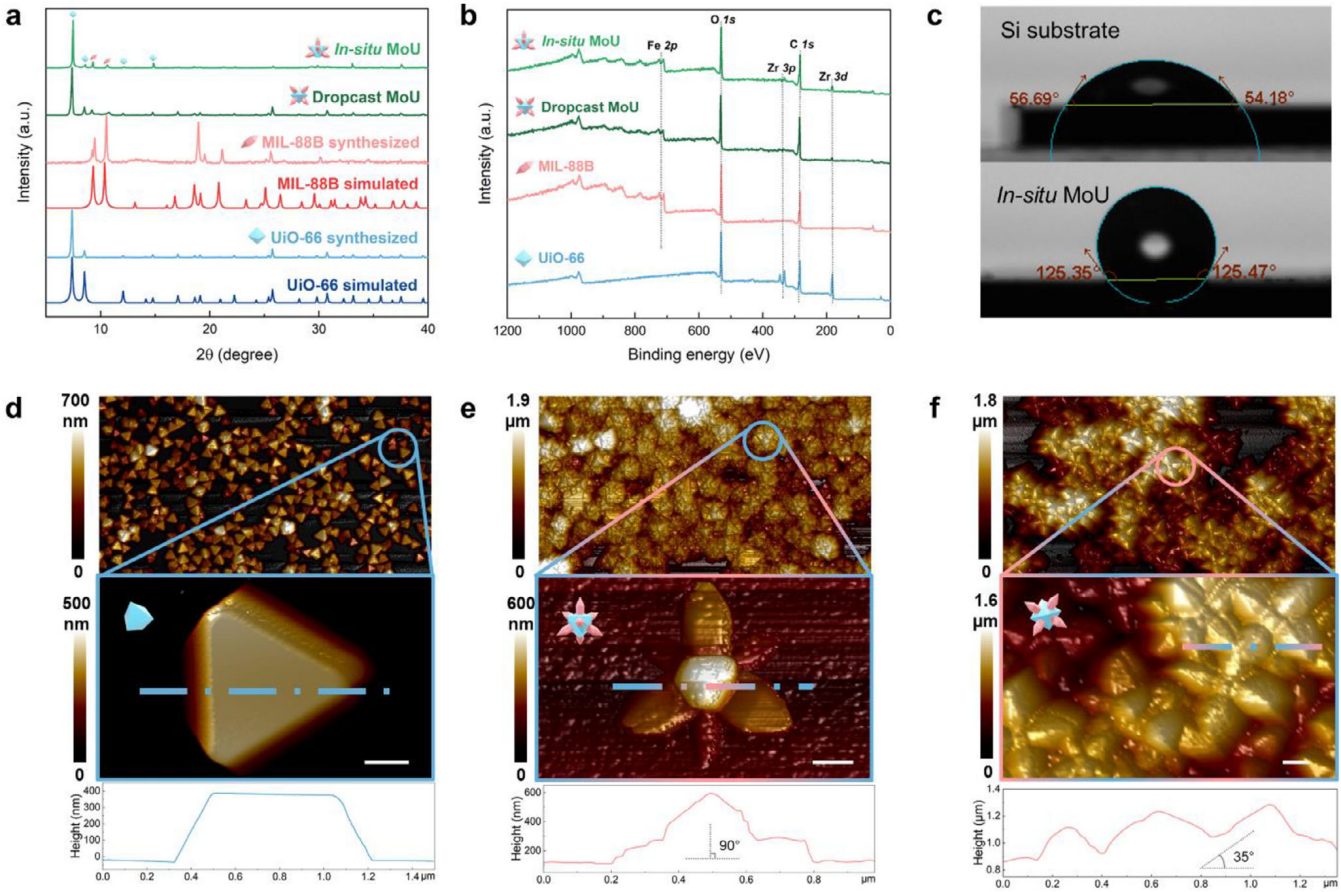
Material characterization of MOF MB surfaces. a) XRD patterns of in situ and dropcast MoU surfaces, UiO‐66, MIL‐88B, and their simulated patterns. The characteristic peaks of UiO‐66 (blue‐octahedron) and MIL‐88B (pink‐rod) are marked at corresponding positions; b) XPS survey spectra of the obtained MOF surfaces with C, O, Fe, and Zr characteristic peaks marked; c) The water contact angle of the silicon substrate and the in situ MoU surfaces showing the enhancement of hydrophobicity after MOF coating. The AFM scanning and cross‐section profile of d) in situ UiO‐66, providing a horizontal plane for MIL‐88B epitaxial growth e) in situ MoU, showing one‐pin up orientation with a near perpendicular MIL‐88B nanopillar, and f) dropcast MoU surfaces, showing four‐pin up orientation with random angles of the MIL‐88B nanopillars, scale bar: 200 nm.

**Figure 3:**
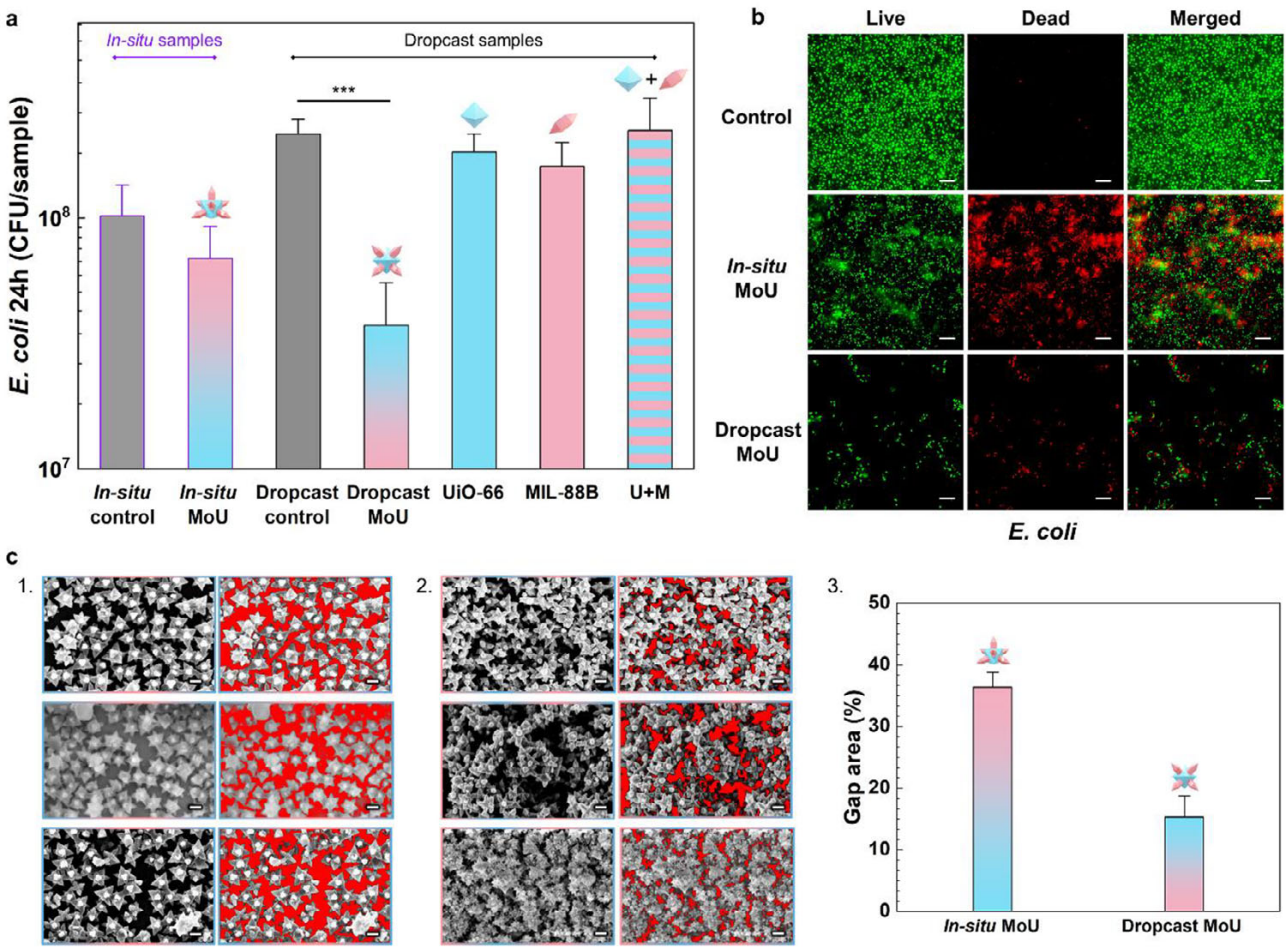
Antibacterial performance of MOF MB surfaces. a) CFU counting results of attached *E. coli* with 24h growth on in situ MoU surfaces and dropcasting surfaces, including MoU, UiO‐66, MIL‐88B, and UiO‐66 + MIL‐88B (U+M). Data represent the mean ± standard deviation of three biological replicates (^***^ *p* < 0.001). b) The live/dead fluorescent staining images of attached E. coli with 24h growth on in situ and dropcasting MoU surfaces, green indicating live bacteria and red indicating dead bacteria, scale bar: 10 µm. c) SEM images of 1) in situ MoU and 2) dropcast MoU surfaces with uncovered areas marked in red, scale bar: 200 nm. 3) The bar chart of the uncovered area percentage of in situ and dropcast MoU surfaces based on three different regions.

To verify their crystal structure and chemical composition, the obtained surfaces were characterized by powder X‐ray diffraction (XRD) and X‐ray photoelectron spectroscopy (XPS). The obtained UiO‐66 and MIL‐88B matched the characteristic peaks of the simulated XRD patterns (**Figure** **2**a; Section S6, Supporting Information), revealing successful MOF synthesis and good crystallinity. Furthermore, in situ and dropcast MoU contained characteristic peaks of UiO‐66 and MIL‐88B, indicating a successful epitaxial MOF‐on‐MOF growth. The XPS survey spectra in Figure 3b show that C, O, Fe, and Zr elements were found on the MoU surfaces, where Fe is from the MIL‐88B nanopillars and Zr from UiO‐66 cores. High‐resolution XPS spectra for Fe *2p* and Zr *3d* confirm the existence of Fe^3+^ and Zr^4+^ in the obtained MoU (Section S7, Supporting Information). SEM/TEM images (Figure 1), XRD and XPS results (Figure 2a,b) jointly confirm the success of MOF‐on‐MOF synthesis in obtaining MoU surfaces, where the nanopillar structures are MIL‐88B(Fe) and the core structures are UiO‐66(Zr).

Surface wettability may influence the attachment propensity of bacteria.^[^ ^49^, ^50^ ^]^ Our in situ MoU surface had the water contact angle (CA) of 127°, compared to the 55° of the intact Si substrates (Figure 2c), indicating increased hydrophobicity. The other MOF surfaces we fabricated were also hydrophobic (Section S8, Supporting Information). The enhancement of the hydrophobicity can likely be attributed to the hydrophobicity of MOFs^[^ ^51^ ^]^ and the nanostructure‐induced surface roughness.^[^ ^52^ ^]^

Atomic force microscopy (AFM) was utilized for mapping the topography of the MOF surfaces. The AFM mapping (Figure 2d–f) confirmed that MOFs as building blocks created spaced nanopatterns. Considering that most parts of the caltrop‐like MOF‐on‐MOF structures are not perpendicular to the substrates, topographical parameters obtained by AFM scanning, such as surface roughness, may not reveal the actual surface features, which are crucial for MB properties of the obtained MOF surfaces. Therefore, we mainly used AFM in this work to understand the orientation differences in the in situ and ex situ MoU surfaces by investigating the zoomed‐in cross‐section profiles for individual MOF structures. As demonstrated in Figure 2d, the in situ grown UiO‐66 provided a near‐horizontal plane for the MIL‐88B to vertically grow epitaxially. Therefore, the MIL‐88B nanopillars in the in situ MoU surface presented a perpendicular orientation to the substrate (Figure 2e), resulting in one‐pin up orientation. By contrast, the MIL‐88B nanopillars issued from the dropcast coating tended to be at ≈35° to the substrate, leading to a four‐pin up orientation. Moreover, due to multilayer stacking, nanopillars in dropcast MoU presented a random orientation (Figure 2f). These findings confirmed that the orientation of the MOF nanopillars can be arranged at different angles by in situ and ex situ methods.

## 3 Mechano‐Bactericidal Actions of MOF Surfaces

MB efficacy of the obtained MOF surfaces was examined by plate counting of colony forming unit (CFU) and live/dead fluorescent staining, using *Escherichia coli* (*E. coli*) and S*taphylococcus epidermidis* (*S. epidermidis*) as model organisms for Gram‐negative and Gram‐positive bacteria, respectively. Additionally, multidrug‐resistant (MDR) *Staphylococcus aureus* (*S. aureus*) was tested as a top priority pathogen. Ruling out chemical cytotoxicity of the MOFs is crucial to the quantitative study of the mechano‐bactericidal performance. Therefore, the zone of inhibition test was carried out for all obtained MOF surfaces. No inhibition zone was found in the MOF surfaces, indicating low chemical cytotoxicity of the MOFs (Section S9, Supporting Information). However, during the tests with liquid culture medium, we observed that MIL‐88B(Fe) presented bactericidal effects toward the Gram‐positive bacteria (both *S. epidermidis* and *S. aureus*). This is most likely caused by Fenton‐like reactions from the iron (Fe) ions released from the MIL‐88B, of which Gram‐positive bacteria are known to be sensitive.^[^ ^53^ ^]^ This intrinsic bactericidal effect of MIL‐88B toward Gram‐positive bacteria impeded the quantitative study of the MB performance of the MOF surfaces. Cumulative effects of chemical toxicity and MB effects on Gram‐positive bacteria are shown in Section S10 (Supporting Information). This left us with *E. coli* as the model for investigating MOF MB effects. Both the in situ MoU and dropcasting MoU surfaces demonstrated MB performance according to the CFU bar chart of attached *E. coli* after 24h growth (**Figure** **3**a; Section S11, Supporting Information). Notably, individual UiO‐66, MIL‐88B, and physical mixture of UiO‐66 + MIL‐88B (denoted as U+M) did not show significant bactericidal efficiency according to their CFU results, which confirmed no/negligible chemical killing effect was involved for *E. coli*. For the in situ MoU surfaces, the bactericidal efficiency was relatively low, at ≈32%. While the bactericidal efficiency of the dropcast MoU surfaces reached ≈83%, landing in the upper tier (over 80%) of the reported solely MB active surfaces (Section S1, Supporting Information). This can probably be explained by the fact that there were fewer uncovered areas on the dropcast MoU surfaces (15%) than on the in situ MoU surfaces (35%), as analyzed by our previously reported image analysis methods (Figure 3c; Section S12, Supporting Information).^[^ ^34^, ^54^ ^]^ The uncovered areas are not effectively protected, as bacteria could attach and survive. This could also be revealed by the live/dead staining images in Figure 3b, where green signals indicate live bacteria and red are dead bacteria. *E. coli* formed a dense biofilm on the control surface, while several isolated bacterial clusters were observed to attach on the in situ MoU surface. For the dropcast MoU surface, separated individual bacteria were observed in the live/dead staining images (Figure 3b), corresponding to a much smaller unprotected area in dropcast surfaces seen in Figure 3c2. The bactericidal efficiency of the dropcast MoU surface dropped from 83% for 24h growth to 51% for 72h growth (Section S13, Supporting Information), which could result from the coverage of the nanostructures by the debris, such as the killed bacteria.^[^ ^55^, ^56^ ^]^ Therefore, an efficient surface cleaning strategy to remove debris could be essential for the long‐term protection and reusability of the MOF MB surfaces.^[^ ^57^, ^58^ ^]^

**Figure 4:**
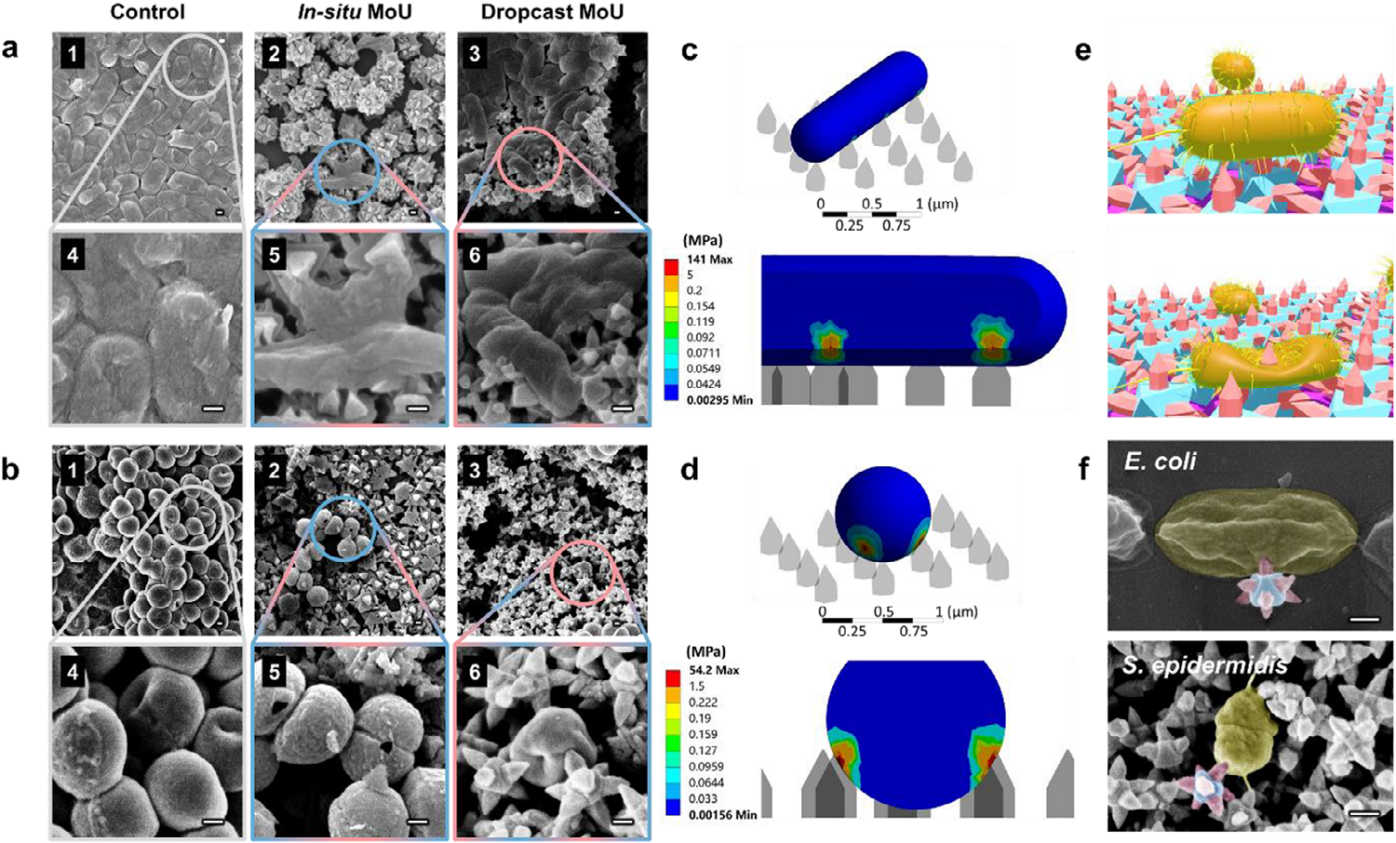
Mechanism study of the MOF MB surfaces. SEM images of attached bacteria on control, in situ MoU, and dropcast MoU surfaces with 24h growth: a) *E. coli*; b) *S. epidermidis*. Stress contour of bacteria on in situ MoU surfaces: c) *E. coli*; d) *S. aureus*. e) Illustration of bacterial rupture by MoU surfaces. f) SEM images of MoU impaling *E. coli* and *S. epidermidis*, false‐colored with bacteria in yellow, UiO‐66 in blue, and MIL‐88B in pink. Scale bar: 200 nm.

To investigate the inactivation mechanisms of the obtained MOF surfaces, the morphology of the bacteria exposed to MOFs was characterized with SEM. As seen in **Figure** **4**a,b, *E. coli* and *S. epidermidis* grew dense biofilms on the control glass surfaces. On the contrary, neither of the bacteria was able to develop a continuous biofilm, and both presented ruptured structures on in situ and dropcast MoU surfaces. Different types of bacterial deformation were observed on the two different MoU surfaces. For instance, cell rupture by *stretching* was found in *E. coli* on in situ MoU surfaces (Figure 4a5), where the *E. coli* cell was pinned by several MOF nanopillars and the bacterial envelope deformed by stretching and tearing.^[^ ^7^ ^]^ Cell rupture by direct *impaling* was found in *E. coli* on dropcast MoU surfaces (Figure 4a6), where MOF nanopillars directly penetrated the bacterial membrane, resulting in deflated morphology and cytoplasm leakage.^[^ ^59^ ^]^ Direct impaling was also observed in *S. epidermidis*, illustrated by triangle‐like holes found on cells exposed to in situ MoU surfaces (Figure 4b5). Apart from stretching and impaling, some bacteria suffered non‐piercing mechanical injury, e.g., *S. epidermidis* on the dropcast MoU surfaces presented a squeezed morphology (Figure 4b6). Even though the MOF nanopillars did not lead to direct penetration, the non‐piercing mechanical injury alone has been reported to induce oxidative stress, ultimately leading to apoptosis‐like death.^[^ ^60^, ^61^ ^]^ Tilted SEM images were acquired to provide close‐up views of the interactions between bacterial cells and MOF structures, where the interfaces of the nanopillars impaling bacterial envelopes were revealed (Section S14, Supporting Information). Similar rupture phenomena were found in MDR *S. aureus* (Section S14 and S15, Supporting Information). Notably, both in situ and ex situ MoU surfaces presented intact structures and surface coverage after antibacterial evaluation, suggesting good structural stability (Section S16, Supporting Information). Understanding the stress distribution on the bacterial cell is essential for understanding the killing mechanism of MB surfaces. Therefore, we conducted a simulation of stress analysis for *E. coli* and *S. aureus* on in situ MoU surfaces (Section S17, Supporting Information). As shown in the stress contour in Figure 4c,d, the maximum stress on the bacterial envelope is 114 mPa for *E. coli* and 54 mPa for *S. aureus*. These maxima exceed the critical elastic stress of the bacteria.^[^ ^62^ ^]^ Since the maximum stress occurs at the areas contacting tips of the nanopillars, the simulation results support the feasibility of the MOF nanopillars puncturing the bacterial membrane, as illustrated in Figure 4e (Video S3, Supporting Information). Two SEM images of MoU directly impaling *E. coli* and *S. epidermidis* with the tip of the nanopillar puncturing the bacterial envelope are shown in Figure 4f.

## 4 Conclusion

In this work, we designed mechano‐bactericidal (MB) surfaces using the epitaxial MOF‐on‐MOF hybrids. The caltrop‐like MIL‐88B‐on‐UiO‐66 (MoU) hybrids were assembled on the surfaces using two approaches: in situ growth and ex situ dropcasting, resulting in one‐pin and four‐pin up orientations of the MoU, respectively. We demonstrated that the features of the MoU surfaces can be rationally controlled to fulfill the key criteria for MB actions: sharp nanostructures with vertical orientation and adequate inter‐spacing. Three types of MB actions were observed, including stretching, impaling, and mechanical injury. Due to low fabrication temperature, a wide range of feasible substrates, the waiver of sophisticated equipment, and the scalability of our MOF‐on‐MOF MB surfaces, we believe that they could make a substantial contribution to the mitigation of the initial attachment of bacteria and slowing down biofilm formation in various applications, such as food packaging, and protection of surfaces on medical implants and devices.

## 5 Experimental Section

### Fabrication of the MOF MB Surfaces

MOFs, including UiO‐66, MIL‐88B, and MoU, were obtained using solvothermal synthesis by modifying reported processes.^[^ ^36^, ^37^, ^39^ ^]^ The in situ UiO‐66 surfaces were obtained by loading the Si chips (6 mm × 6 mm) into the autoclave during UiO‐66 synthesis. In situ MoU surfaces were obtained by loading the in situ UiO‐66 surfaces into the Pyrex tube during MoU synthesis. Dropcast MOF surfaces were obtained by dropcasting 50 µL MOF solution (5 mg mL^−1^ in ethanol) on the round glass slide (diameter 10 mm). All the samples were dried in a static vacuum oven at 60 °C overnight before tests. More details on the MOF synthesis and fabrication of the MOF MB surfaces are described in Section S18 (Supporting Information).

### Scanning Electron Microscopy (SEM)

The microstructure of the samples was characterized by SEM (Zeiss Supra 55VP) with 5 kV accelerating voltage. The samples containing bacteria were fixed with 3% glutaraldehyde for 2 h and then dehydrated by a series of ethanol concentrations (40%, 50%, 60%, 70%, 80%, and 90%) for 10 min each, and with absolute ethanol for 15 min. All samples were dried in a static vacuum oven for 3 h at 60 °C and coated with a 15 nm gold layer to avoid charging.

### Transmission Electron Microscopy (TEM)

The TEM images were acquired from an FEI Tecnai T20 microscope, equipped with a LaB6 filament and operating at 200 kV accelerating voltage. The MOF solution (1 mg mL^−1^ in ethanol) was dropcast on carbon‐film supported copper grids (Sigma–Aldrich) and dried in a static vacuum oven for 3 h at 60 °C before tests.

### X‐Ray Diffraction (XRD)

The powder X‐ray diffraction (PXRD) was characterized by using a Bruker XRD D8 Advance with Cu Kα X‐ray source (λ = 1.54 Å) at room temperature, with a scanning range 2θ from 5° to 40°. Simulated PXRD patterns were calculated with Mercury software with crystal data from the Cambridge Structure Database (CSD).

### X‐Ray Photoelectron Spectroscopy (XPS)

The surface chemistry of the obtained MOF samples was studied with a PHI VersaProbe III X‐ray photoelectron spectroscopy (XPS) instrument with an Al Kα X‐ray source. The data analysis was performed with CasaXPS software.

### Contact Angle (CA) Measurements

The wettability of the obtained surfaces was characterized by measuring the water contact angle in the air using an optical tensiometer (Attension, Biolin Scientific). The images were taken within 5 s of the droplets being dispensed on the surfaces. Statistical averaging of 3 replicates was performed on each sample.

### Atomic Force Microscopy (AFM)

The surface topography was studied using atomic force microscopy using Dimension ICON (Bruker) with tapping mode with Bruker RTESP‐300 AFM probes. All samples were coated with a 15 nm gold layer to reduce the risk of picking MOF particles during AFM measurement.

### Computational Stress Analysis

To study the stress of the bacterial envelope on the MOF surfaces, commercial software (ANSYS 2024 R1) was used as a finite element analysis environment. The shape of *E. coli* and *S. aureus* bacteria was modeled as a spherocylinder and sphere, respectively. The geometry parameters of the bacterial size and MOF size were obtained from the SEM and TEM images. Other parameters, including Young's modulus of the bacterial envelope and the adhesion forces between bacteria and substrates, were collected from reported literature. All parameters used for the simulation were summarized in Section S17 (Supporting Information).

### Evaluation of Bacterial Viability

The antibacterial performance was evaluated through plate counting of colony‐forming unit (CFU) method as reported in our previous studies.^[^ ^17^, ^35^ ^]^ The bacterial strains *Escherichia coli* (UTI89), *Staphylococcus epidermidis* (ATCC 35984), and *Staphylococcus aureus* (CCUG 35571) were obtained from Gothenburg University Culture Collection (CCUG) and used for antibacterial evaluation on the obtained surfaces. Single colonies of each bacterial strain were grown in a liquid medium (5mL), Luria‐Bertani (LB) broth for *E. coli* and tryptic soy broth (TSB) for *S. epidermidis* and *S. aureus*, at 37°C overnight. Then, 25 µL overnight bacterial culture was added to 5 mL fresh medium to obtain an inoculum containing 2–5 × 10^6^ CFU mL^−1^ bacteria, where the bacterial cell density was verified by plate counting. The inoculum was then loaded onto the tested surfaces, at 40 and 100 µL to the silicon substrated (square, 6 mm × 6 mm) samples and glass slide substrated (round, diameter 10 mm) samples, respectively, to make sure the MOF surfaces fully covered while not contaminating the uncoated surfaces. Notably, to avoid the evaporation of the culture media on the sample surfaces, the tested samples were placed in the middle part of a 24‐well plate, while the surrounding empty wells at the edges, as well as the gaps of the plate, were filled with sterilized distilled water. After 24 h bacterial growth at 37 °C, the media culture was removed from the surface, and irreversibly attached bacteria were detached from surfaces by sonication (Digital Sonifier, Branson, 10% amplitude, 30 s) and collected in saline solution (5 mL of 0.89% NaCl). Thereafter, the collected bacteria were diluted (×10) serially and plated in agar plates. The plates were incubated at 37 °C for 24h. The number of colonies on the collected plates was counted, and the number of viable bacteria (CFU sample^−1^) was then estimated by the number of colonies counted in plates and their corresponding dilution factors. The bactericidal efficiency was obtained by normalizing the CFU counts of each surface corresponding to that of the control surface. The experiments were conducted with three biological replicates, and the mean values ± standard deviation were reported. The statistical significance between the MoU surface and control surface was examined using an independent *t*‐test at *p* ≤ 0.05.

### Fluorescence Microscopy

The live/dead assay was done with fluorescence microscopy. The attached bacteria on the surface were stained with LIVE/DEAD BacLight bacteria viability stains kit L7012 (Invitrogen, Molecular Probes, Inc. Eugene, OR, USA). The kit consists of the green‐fluorescent nucleic acid stain SYTO 9 and the red‐fluorescent nucleic acid stain propidium iodide (PI). The green‐fluorescent dye (SYTO 9) crosses all bacterial membranes and binds to the DNA of bacterial cells. The red‐fluorescent PI only crosses damaged bacterial membranes (dead bacteria). Fluorescence microscopy imaging of the attached bacteria was acquired using a fluorescence microscope (LeicaDMi8) with a Z‐stack function, after staining the samples with a mixture of SYTO 9 and PI for 20 min. Experiments were performed in three biological replicates, and representative images were presented.

## Conflict of Interest

The authors declare no conflict of interest.

## Author Contributions

Z.C. contributed to the conceptualization, methodology, formal analysis, investigation, data curation, original draft writing, review and editing, visualization, and funding acquisition. S.P. was involved in methodology, formal analysis, investigation, data curation, review and editing, supervision, and funding acquisition. F.M.A.N. contributed to methodology, investigation, review and editing, supervision, and funding acquisition. J.Z. participated in methodology, formal analysis, data curation, and review and editing. W.G. contributed to methodology, formal analysis, data curation, and review and editing. S.R. was involved in methodology and review, and editing. L.Ö. contributed to conceptualization, methodology, formal analysis, investigation, data curation, review and editing, supervision, project administration, and funding acquisition. I.M. was responsible for conceptualization, methodology, formal analysis, investigation, data curation, review and editing, supervision, project administration, and funding acquisition.

## Supporting information



Supporting Information

Supplemental Video 1

Supplemental Video 2

Supplemental Video 3

## Data Availability

The data that support the findings of this study are available from the corresponding author upon reasonable request.
