# Supplementary material for: Mechano‐Bactericidal Surfaces Achieved by Epitaxial Growth of Metal–Organic Frameworks
Source: Adv Sci (Weinh). 2025 Aug 30;12(46):e05976. doi: 10.1002/advs.202505976 (PMC12697766; doi:10.1002/advs.202505976)
Supplement: Supplementary file 1 — Supporting Information [file ADVS-12-e05976-s001.pdf]

Supporting Information

Mechano-Bactericidal Surfaces Achieved by Epitaxial Growth of Metal-Organic Frameworks

Zhejian Cao, Santosh Pandit, Francoise M. Amombo Noa, Jian Zhang, Wengeng Gao, Shadi Rahimi, Lars Öhrström, and Ivan Mijakovic

E-mail: zhejian@chalmers.se, ohrstrom@chalmers.se, ivan.mijakovic@chalmers.se

Contents

Section 1: Natural and artificial mechano-bactericidal surfaces 2
Section 2: Antibacterial metal-organic frameworks by metal ion release 3
Section 3: Spacing analysis of the *in-situ* MIL-88B-on-UiO-66 (MoU) surfaces 4
Section 4: Feasibility of modifying the MOF surface features 5
Section 5: Dropcast MoU hybrids on different substrates 6
Section 6: Simulated XRD patterns of UiO-66 and MIL-88B 7
Section 7: High-resolution XPS spectra for Fe *2p* and Zr *3d* 8
Section 8: Water contact angle measurement 9
Section 9: Zone of inhibition test 10
Section 10: Bactericidal efficiency for Gram-positive bacteria 11
Section 11: Representative plate photographs of CFU counting 12
Section 12: Effective bactericidal area analysis 13
Section 13: Bactericidal efficiency of dropcast MoU surface for 72h 14
Section 14: Tilted SEM images of *in-situ* MOF MB surfaces 15
Section 15: SEM images of attached bacteria on MOF surfaces 16
Section 16: Stability of the MoU hybrid and MoU surfaces 17
Section 17: Stress analysis simulation 18
Section 18: Fabrication of MOF MB surfaces 19
Reference for supplementary information 20

Section 1: Natural and artificial mechano-bactericidal surfaces

The natural and artificial mechano-bactericidal (MB) surfaces are summarized in **Table S1**.

Table S1 Summary of natural and artificial mechano-bactericidal surfaces

Surfaces	Preparation method	Surface features (height; tip/base diameter; pitch)	Wettability (water contact angle (CA))	Tested species	Bactericidal activity	Refs
Natural mechano-bactericidal surfaces						
Cicada wings (*P. claripennis*)	Natural	200 nm; 60 nm / 100 nm; 170 nm	Hydrophobic, CA = 158°	*P. aeruginosa*	Lethal in 3 min	[1]
Dragonfly wing (*D. bipunctata*)	Natural	240 nm; 50 nm; NA	Hydrophobic, CA = 153°	*P. aeruginosa, S. aureus, B. subtilis*	lethal effect to all 3 bacteria in 3 h	[2]
Gecko skin (*L. steindachneri*)	Natural	up to 4 µm; tip curvature in the range of 10–30 nm; sub-micron spacing	Hydrophobic, CA = 151-155°	*P. gingivalis*	Lethal effect up to 7 days	[3]
Gecko skin (*S. williamsi*)	Natural	3 µm; 50 nm / 350-450 nm; 500 nm	Hydrophobic, CA = 134°	*Co-culture of 7 gut bacteria*	bactericidal function observed	[4]
Cicada wings (*M. intermedia*)	Natural	241 nm; 156 nm; 165	Hydrophobic, CA = 136°	*P. fluorescens*	Active at killing	[5]
Cicada wings (*C. aguila*)	Natural	182 nm; 159 nm; 187 nm	Hydrophobic, CA = 113°	*P. fluorescens*	Active at killing	[5]
Cicada wings (*Magicicada ssp.*)	Natural	84 nm; 167 nm; 252 nm	Hydrophilic, CA = 80°	*S. cerevisiae (Yeast)*	Cell wall rupture	[6]
Cicada wings (*Tibicen ssp.*)	Natural	183 nm; 57 nm / 104 nm; 175 nm	Hydrophobic, CA = 132°	*S. cerevisiae (Yeast)*	Cell wall rupture	[6]
Dragonfly wings (*Progomphus. ssp.*)	Natural	241 nm; 53 nm; 123 nm	Hydrophobic, CA = 119°	*S. cerevisiae (Yeast)*	Cell wall rupture	[6]
Amorpha fruticosa leaf	Natural	~1 µm; randomly arranged	Hydrophobic, CA = 154°	*E. coli*	Cell rupture	[7]
Artificial mechano-bactericidal surfaces (top-down approach: TD; bottom-up approach: BU)						
Black silicon (TD)	Reactive-ion etching (RIE)	280 nm; 62 nm; 62 nm	Hydrophilic, CA = 8°	*P. aeruginosa, S. aureus*	89% for *P. aeruginosa;* 85% for *S. aureus in* 24 h	[8]
Black silicon (TD)	RIE	500 nm; 20-80 nm; NA	Hydrophilic, CA = 80°	*P. aeruginosa, S. aureus, B. subtilis*	lethal effect to all 3 bacteria in 3 h	[2]
Black silicon (TD)	RIE	NA; 150-200 nm; 100-250 nm	NA	*E. coli, S. aureus, B. cereus*	over 99% in 24h for all 3 bacteria	[9]
Black silicon (TD)	RIE	4 µm; 10-20 nm / 220 nm; NA	Hydrophobic, CA = 154°	*E. coli, S. aureus*	83% for *E. coli* and 86% for *S. aureus* in 3 h	[10]
Graphene nanosheets (BU)	Exfoliation + filtration	horizontal length 80 nm; width 5 nm; NA	NA	*P. aeruginosa, S. aureus*	71% for *P. aeruginosa* and 77% for *S. aureus*	[11]
Graphene nanosheets (BU)	Chemical vapor deposition (CVD)	60-100 nm; width less than 5 nm; NA	Hydrophobic, CA = 117°	*E. coli, S. epidermidis*	over 85% for *E. coli* and over 90% for *S. epidermidis* in 4 h	[12]
Carbon nanotubes (BU)	CVD	1 µm; 50 nm; less than 10 nm	Hydrophobic, WCA = 149°	*P. aeruginosa, S. aureus*	99.3% for *P. aeruginosa* and 84.9% for *S. aureus*	[13]
Titania (TD)	Hydrothermal etching	3 µm; 100 nm; NA	NA	*P. aeruginosa, E. coli, S. aureus*	over 40% for *P. aeruginosa,* over 80% for *E. coli;* 5% for *S. aureus*	[14]
Titania (TD)	Hydrothermal etching	NA; 40 nm; NA	Hydrophilic, CA = 73°	*P. aeruginosa, S. aureus*	47% for *P. aeruginosa* and 20% for *S. aureus*	[15]
Titania (TD)	RIE	1 µm; 80 nm; NA	Hydrophilic, CA = 10°	*E. coli, P. aeruginosa, M. smegmatis, S. aureus*	95% for *E. coli*, 98% for *P. aeruginosa*, 92% for *M. smegmatis*, and 22% for *S. aureus* in 4h	[16]
Titania (TD)	Hydrothermal etching	4 µm; 50 nm / 100 nm; 3-5 µm	Hydrophilic, CA = 0°	*S. epidermidis*	47% killing for *S. epidermidis*	[17]
Titania (BU)	Anodization	2 µm; 10-30 nm; 2 µm	Hydrophilic, CA = 0°	*S. aureus*	10-fold reduction	[18]
Gold (BU)	Anodization deposition	100 nm; 50 nm; NA	NA	*S. aureus*	99% for *S. aureus*	[19]
Ormostamp polymer (TD)	Templates by anodization and deep RIE	150 nm - 400 nm; 80nm; 130 - 300 nm	NA	*S. aureus*	over 99%	[20]
PMMA (TD)	Stamping with molds	210-300 nm; 70-215 nm; 100-380 nm	NA	*E. coli*	over 50%	[21]
**MOF-on-MOF (BU)**	**Solvothermal synthesis**	**300 nm; ~ 5 nm / 200 nm; ~ 500 nm**	**Hydrophobic, CA = 127°**	***E. coli, S. epidermidis, S. aureus***	**83% for *E. coli;* 70% for *S. epidermidis;* 99% for *S. aureus* (synergetic effect for Gram-positive bacteria)**	**This work**

Section 2: Antibacterial metal-organic frameworks by metal ion release

The antibacterial metal-organic frameworks (MOFs) by metal ion release are summarized in **Table S2**.

Table S2 Summary of antibacterial MOFs by metal ion release

MOFs	Antibacterial mechanism proposed	Tested species	Bactericidal activity	Refs
$Ag_2(O\text{-}IPA)(H_2O)\cdot(H_3O)$	Ag ion release	*E. coli, S. aureus*	*E. coli*: MIC = 5–10 ppm; *S. aureus*: MIC = 10–15 ppm	[22]
$Ag_5(PYDC)2(OH)$	Ag ion release	*E. coli, S. aureus*	*E. coli*: MIC = 10–15 ppm; *S. aureus*: MIC = 15–20 ppm	[22]
Ag_3(3-phosphonobenzoate)	Ag ion release	*P. aeruginosa, E. coli, S. aureus*	*P. aeruginosa*: MBC = 20–30 µM; *E. coli*: MBC = 50 µM; *S. aureus*: MBC = 50–75 µM	[23]
$[(AgL)NO_3]\cdot 2H_2O$	Ag ion release	*E. coli, S. aureus*	*E. coli*: MIC = 300 µM; *S. aureus*: MIC = 297 µM	[24]
$[(AgL)CF_3SO_3]\cdot 2H_2O$	Ag ion release	*E. coli, S. aureus*	*E. coli*: MIC = 300 µM; *S. aureus*: MIC = 307 µM	[24]
$[(AgL)ClO_4]\cdot 2H_2O$	Ag ion release	*E. coli, S. aureus*	*E. coli*: MIC = 308 µM; *S. aureus*: MIC = 293 µM	[24]
$[Ag_2(\mu\text{-}PTA)_2(\mu\text{-}suc)]_n\cdot 2nH_2O$, $[Ag_2(\mu\text{-}PTA)_2(\mu_4\text{-}adip)]_n\cdot 2nH_2O$, $[Ag_2(\mu_4\text{-}PTA)(\mu_4\text{-}mal)]_n$	Ag ion release	*P. aeruginosa, E. coli, S. aureus, C. albicans*	*P. aeruginosa*: MIC = 6-20 µM; *E. coli*: MIC = 6-7 µM; *S. aureus*: MIC = 6-40 µM; *C. albicans*: MIC = 6-40 µM	[25]
$[Ag_7(bte)_4(H_2O)\ (HP_2W^{VI}_{16}W^{V}_2O_{62})]\cdot 2H_2O$, $[Ag_7(btp)_5(HP_2W^{VI}_{16}W^{V}_2O_{62})]\cdot H_2O$, $[Ag_4(btb)_{3.5}(P_2W_{18}O_{62})](H_2btb)\cdot 2H_2O$	Ag ion release	*E. coli, S. aureus*	Inhibition zone observed	[26]
CuBTC (HKUST-1)	Cu ion release	*E. coli, S. aureus*	Inhibition zone observed	[27]
CuBTC (HKUST-1)	Cu ion release	*E. coli*	Inhibition zone observed	[28]
CuBTC (HKUST-1)	Cu ion release	*S. cerevisiae, G. candidum*	*S. cerevisiae*: inhibition of growth in 24h, *G. candidum*: over 99% reduction in 24h	[29]
Cu-SURMOF 2	Cu ion release	Cobetia marina	over 99% bacteria killed in 2h	[30]
$n[Cu(AIP)_2(PIY)(H_2O)_2]\cdot 4H_2O$	Cu ion release	*P. aeruginosa, E. coli, Candida spp., S. aureus, Klebsiella sp.*	Inhibition zone observed	[31]
MPN-DMOF*	Cu ion release	*P. aeruginosa, B. vietnamensis, E. coli, and S. aureus*	1.6, 1.5, 0.9, and 1.1-log CFU reduction in the mature biofilms	[32]
ZnBDC (MOF-5)	Zn ion release	*P. aeruginosa*	*P. aeruginosa*: MIC = 25 µM	[33]
$[Zn(bipy)(OH_2)_4^{2+}]_{1.5}[ClO4^-]_3\cdot(bipy)_3(H_2O)$	Zn ion release	*E. coli, S. epidermidis*	*E. coli*: MIC = 5.3 ppm; *S. epidermidis*: MIC = 3.8 ppm	[34]
$[Zn_{1.5}(CH_3CO_2)_2(bipy)^{2+}][ClO4^-]\cdot H_2O$	Zn ion release	*E. coli, S. epidermidis*	*E. coli*: MIC = 6.1 ppm; *S. epidermidis*: MIC = 4.6 ppm	[34]
Zn(Im)2 (ZIF-4)	Zn ion release	*E. coli, S. aureus*	Inhibition zone observed for *S. aureus*	[35]
Zn(bIm)2 (ZIF-7)*	Zn ion release	*E. coli, S. aureus*	Inhibition zone observed for *S. aureus*	[35]
Zn(MeIm)2 (ZIF-8)	Zn ion release	*E. coli, S. aureus*	Inhibition zone observed	[35]
BioMIL-5	Zn ion release	*S. aureus, S. epidermidis*	*S. aureus*: MIC =1.7 mg/mL; *S. epidermidis*: MIC =1.7 mg/mL	[36]
Co-TDM	Co ion release	*E. coli*	MBC=10-15 ppm	[37]
ZIF-67	Co ion release	*E. coli; P. putida; S. cerevisiae*	Inhibition zone observed for both *E. coli and P. putida*	[38]
Co-SIM-1	Co ion release	*E. coli; P. putida; S. cerevisiae*	Inhibition zone observed for both *E. coli and P. putida*	[38]

*Some studies discussed the synergistic antimicrobial mechanisms of physical damage and other means, including positive charge[39]/cation release[32]. However, the quantitative contribution from the mechano-bactericidal part remains elusive. Furthermore, rational control of the surface features was not covered compared to the typical MB surfaces, such as tip size less than 200 nm, as summarized in **Table S1**.

Section 3: Spacing analysis of the *in-situ* MIL-88B-on-UiO-66 (MoU) surfaces

The spacing/pitch distance of the nanostructures is one of the critical surface features for MB surfaces. The spacing of the vertical MIL-88B nanopillars is controlled by the distance between the center of UiO-66 cores, as the MIL-88B grew epitaxially from the UiO-66. The spacing between the adjacent MIL-88B nanopillars and UiO-66 cores in the *in-situ* surfaces is analyzed using ImageJ software. The results show that the spacing of MIL-88B nanopillars and UiO-66 cores are approximately 500 nm (527 ± 120 nm for MIL88-B and 542 ± 174 nm for UiO-66), as shown in **Table S3** and **S4**, **Figure S1** and **S2**.

Table S3 Spacing between vertical MIL-88B nanopillars

Number	Length (nm)	Number	Length (nm)
1	462	16	632
2	415	17	597
3	533	18	425
4	577	19	456
5	496	20	545
6	543	21	720
7	447	22	555
8	405	23	715
9	597	24	495
10	273	25	234
11	562	26	523
12	544	27	623
13	526	28	797
14	610	**Mean**	**527**
15	441	**Standard deviation**	**120**

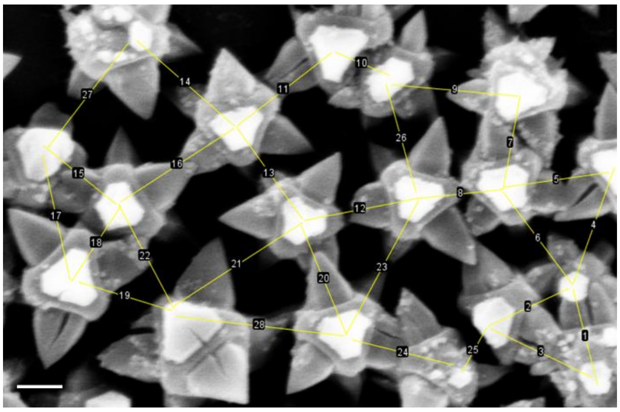

Figure S1 Spacing analysis of *in-situ* MoU surface, scale bar 200 nm

Table S4 Spacing between UiO-66 cores

Number	Length (nm)	Number	Length (nm)
1	517	21	902
2	547	22	1054
3	570	23	454
4	271	24	489
5	222	25	383
6	432	26	569
7	643	27	623
8	482	28	629
9	437	29	245
10	412	30	547
11	549	31	401
12	425	32	735
13	715	33	475
14	468	34	483
15	367	35	475
16	756	36	771
17	561	37	725
18	427	38	836
19	523	**Mean**	**542**
20	469	**Standard deviation**	**174**

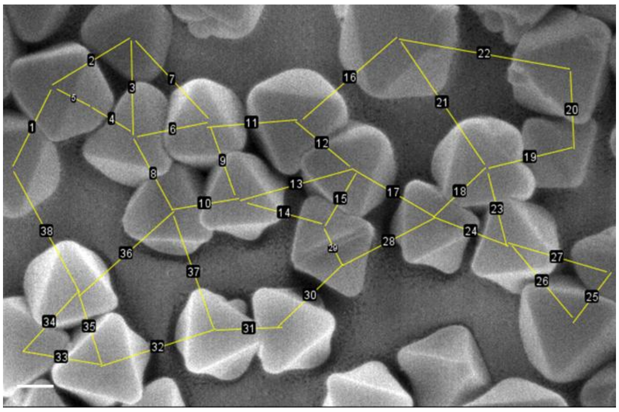

Figure S2 Spacing analysis of *in-situ* UiO-66 surface, scale bar 200 nm

Section 4: Feasibility of modifying the MOF surface features

The surface features of the MB surfaces are essential to the MB performance.[40,41] One advantage of using MOF as the building block for MB surfaces is that there are various strategies to tune the geometry parameters of MOFs, such as adjusting the synthesis conditions.[42] More specifically, modulators play an important role in the nucleation and growth of MOF crystals.[43,44] By tuning the ratio of the modulator (in this study, acetic acid for UiO-66), we adjusted the size of the UiO-66 and achieved different diameters and lengths of the nanospikes in *in-situ* MoU surfaces as shown in **Figure S3a**. The pitch distance of the *in-situ* MoU is controlled by the *in-situ* UiO-66 cores, as discussed in **SI Section 3**. Many approaches to achieving a dense UiO-66 film were reported, such as adjusting the concentration of the modulator,[43] and repetition of the solvothermal treatments.[45] We have performed both methods to achieve a dense UiO-66 surface, as shown in **Figure S3bc**. The pitch distance and uncovered area could be reduced. However, using the repetition of solvothermal treatment (twice) might produce UiO-66 particles with different sizes (**Figure S3c**), resulting in a large standard deviation in the pitch distance. Our results verified the feasibility of rationally designing the surface features of the MOF MB surfaces, including pitch distance, diameter, and length of the nanospikes. However, precise control of the surface features requires systematic study with different synthesis conditions. Furthermore, an in-depth comprehensive study on the relationship between the geometry parameters of MOF surfaces and their bactericidal performance would be an important forward research direction, especially considering different bacteria possessing different geometries.

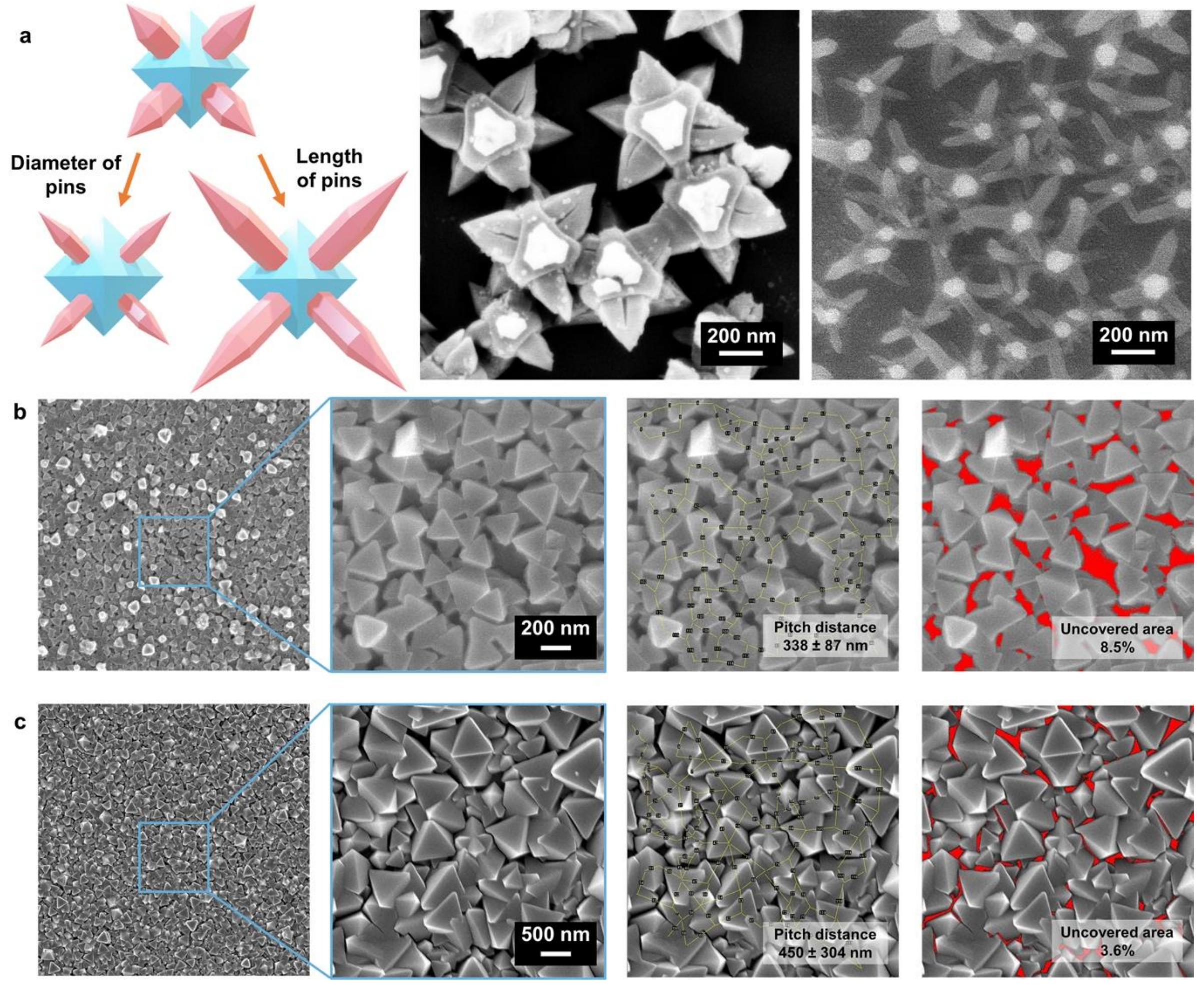

Figure S3 (a) Illustration and SEM images of the *in-situ* MoU surface with different diameters and lengths of the MIL-88B nanopillars; (b) dense *in-situ* UiO-66 surface achieved by tuning the modulator ratio; (c) dense *in-situ* UiO-66 surface achieved by two repetitions of solvothermal treatment

Section 5: Dropcast MoU hybrids on different substrates

The melting point of typical medical plastics[46] and other materials[47] were summarized in **Table S5**. Both *in-situ* growth (120 °C) and *ex-situ* dropcasting (room temperature) methods work below the melting point of the common medical materials. Through an *ex-situ* method, MOF nanostructures can be loaded on various substrates. For instance, the MoU hybrids were loaded on different substrates by dropcasting as shown in **Figure S4**. Four substrates including glass, stainless steel, wood, and polyethylene terephthalate (PET) slice have been used as the substrates. Moreover, pilot-scale production of UiO-66(Zr) has been reported with over 93% yield,[48] and the production of MIL-88B(Fe) has been scaled up by replacing DMF with ethanol.[49] The low fabrication temperature of MB surface assembly and reported scale-up cases indicate promising potential for large-scale production of MoU MB surfaces on different substrates. Even though the heterogeneity of the MOF coating was observed (**Figure S4, glass sample**), the surfaces were fully covered with MOFs with current MOF loading (5 mg/mL, 50 μL). Therefore, these surfaces are suitable for the antibacterial evaluation in this study, as the MB interactions between the MOFs and bacteria occur on the surface of the nanostructures. To achieve a more homogenous surface of the MOFs on the surfaces, some other *ex-situ* methods could be applied in future studies, such as spin coating.[50]

Table S5 Melting temperature of common medical materials

Materials		Melting temperature (°C)
**Polymers**	High-density polyethylene (HDPE)	134
	Polypropylene (PP)	170
	Polyvinyl chloride (PVC)	170
	Polyamides (Nylon 6)	215
	Polyethylene terephthalate (PET)	255
	polytetrafluorethylene (PTFE)	327
**Metals**	Gold	1064
	Stainless steel (316)	1375
	Titanium	1668
**Ceramics**	Glass	1400
	Aluminum oxide	2072
	Zirconium oxide	2715

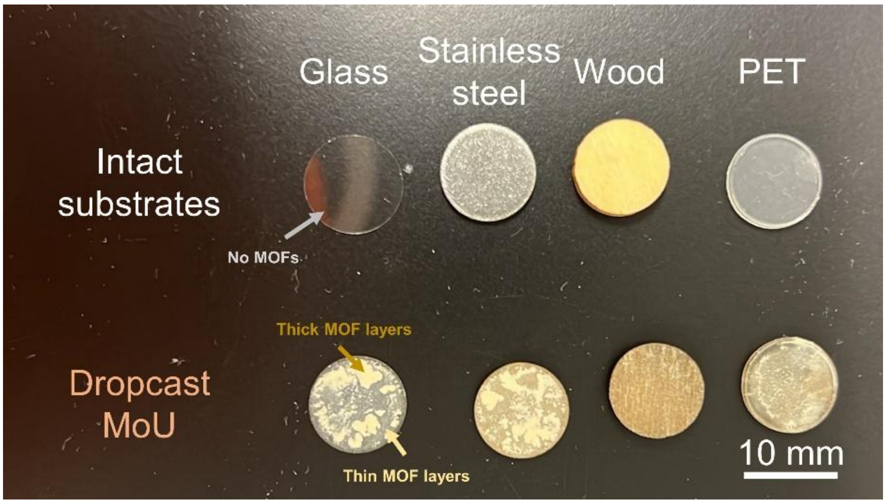

Figure S4 Dropcast MoU on different substrates. The first row is the intact substrates, and the second row is the samples with dropcast MoU. The substrates are from left to right: glass, stainless steel, wood, and PET slice.

Section 6: Simulated XRD patterns of UiO-66 and MIL-88B

The original structure Crystallographic Information Files (CIF) were obtained from Cambridge Crystallographic Data Centre “CCDC” with identifiers “RUBTAK04” and “YEDKOI” for UiO-66 and MIL-88B, respectively. Their structures and XRD patterns are illustrated in **Figure S5**. The XRD patterns were simulated in Mercury software.

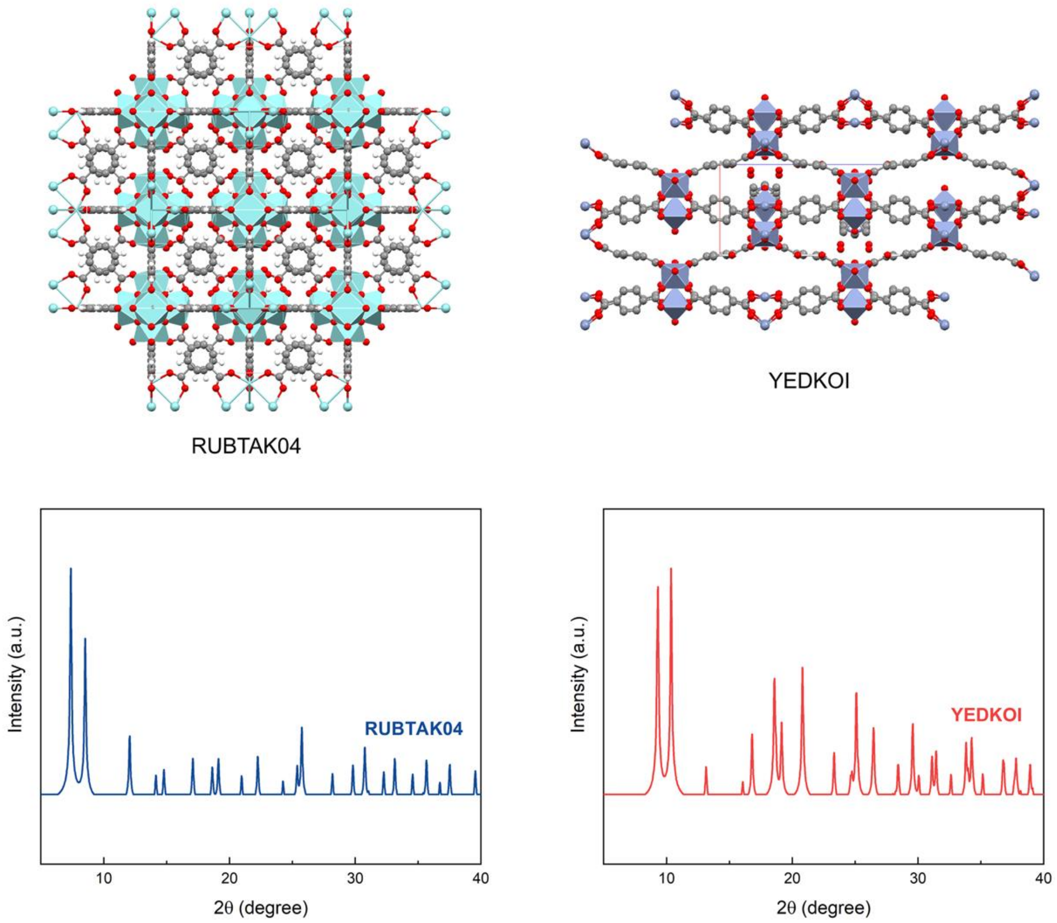

Figure S5 Structures and XRD patterns of RUBTAK04 (UiO-66) and YEDKOI (MIL-88B)

Section 7: High-resolution XPS spectra for Fe *2p* and Zr *3d*

The high-resolution XPS spectra for Fe *2p* and Zr *3d* are shown in **Figure S6**. Fe *2p* peaks were observed in the MIL-88B, *in-situ* MoU, and dropcast MoU samples, as shown in **Figure S6a**. The main Fe *2p* peaks centered at 725.3 eV and 712.0 eV, corresponding to Fe $2p_{1/2}$ and Fe $2p_{3/2}$, respectively.[51] Two satellite (sat.) signals were observed near 731.1 eV and 717.5 eV. These Fe *2p* peaks confirmed the existence of Fe^{3+}.[52] Zr *3d* peaks were observed in the UiO-66, *in-situ* MoU, and dropcast MoU samples, as shown in **Figure S6b**. The main Zr *3d* peaks centered at 184.9 eV and 182.6 eV, corresponding to Zr $3d_{3/2}$ and Zr $3d_{5/2}$, respectively. These Zr *3d* peaks confirmed the existence of Zr^{4+}.[53]

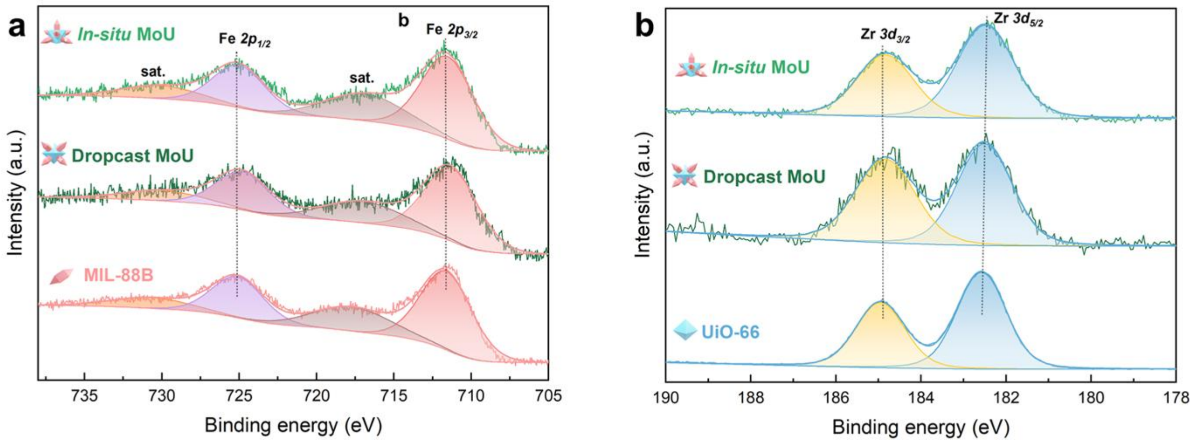

Figure S6 XPS spectra of (a) Fe *2p* and (b) Zr *3d*

Section 8: Water contact angle measurement

The wettability of the obtained surfaces was analyzed by water contact angle (CA) measurement within 5s once the water contacted the surfaces. The hydrophobicity of the surface was enhanced when introducing MOFs, as shown in **Table S6** and **Figure S7**.

Table S6 Water contact angle of obtained surfaces

Number	Si substrate			Glass substrate				
	Si	*In-situ* UiO-66	*In-situ* MoU	Glass	Dropcast UiO-66	Dropcast MIL-88B	Dropcast MIL-88B + UiO-66	Dropcast MoU
1	56	99	126	60	129	124	124	125
2	55	102	129	61	121	125	124	122
3	54	105	125	63	121	123	126	126
Mean	55	102	127	61	124	124	125	124

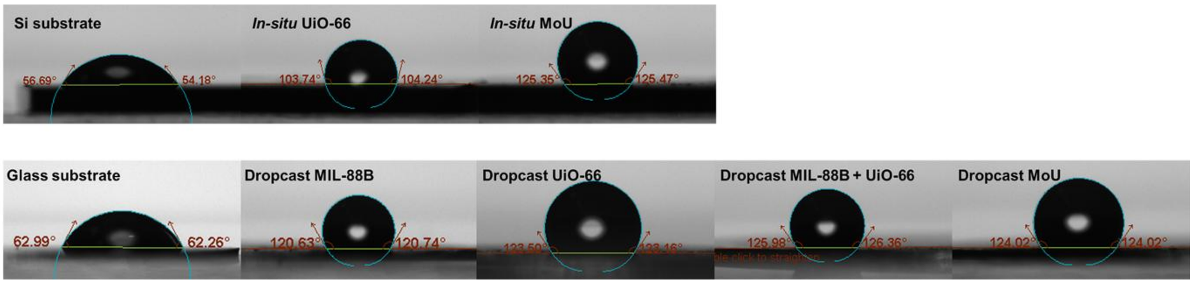

Figure S7 Images of water contact angle measurements for obtained surfaces

Section 9: Zone of inhibition test

The zone of inhibition test was carried out with *E. coli* and *S. aureus* in three biological replicates to preliminarily evaluate the potential chemical leaching and chemical antibacterial activities. Ampicillin was used at the same loading concentration as MoU as a demonstration of the zone of inhibition. As shown in **Table S7** and **Figure S8**, no inhibition zone was observed for obtained MOF surfaces.

Table S7 Zone of inhibition results for the tested MOF surfaces

Time	ZOI with *E. coli* (mm)					ZOI with *S. aureus* (mm)				
	Si	MoU	UiO-66	MIL-88B	Ampicillin	Si	MoU	UiO-66	MIL-88B	Ampicillin
12 h	0	0	0	0	26	0	0	0	0	45
24 h	0	0	0	0	26	0	0	0	0	50
48 h	0	0	0	0	26	0	0	0	0	51
72 h	0	0	0	0	26	0	0	0	0	51

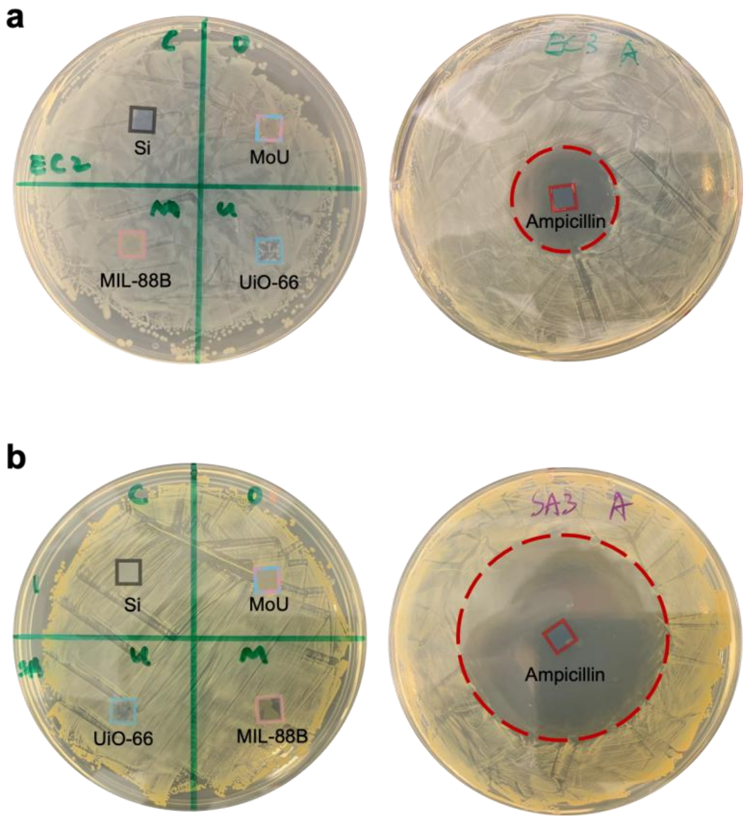

Figure S8 Images of zone of inhibition (ZOI) test with 24h growth of (a) *E. coli* and (b) *S. aureus;* the boundaries of tested samples were highlighted with square frames; ZOI was found in ampicillin samples, marked with dashed circles

Section 10: Bactericidal efficiency for Gram-positive bacteria

The antibacterial performance of the MOF MB surface towards Gram-positive bacteria was evaluated by colony forming unit (CFU) counting method and live/dead staining, as shown in **Figure S9**. The surfaces containing MIL-88B presented a considerable reduction in the CFU counting. For example, dropcast MIL-88B demonstrated 97.7% and 99.9% bactericidal efficiency for *S. epidermidis* and *S. aureus*, respectively. This excellent bactericidal efficiency was not only owing to the mechano-bactericidal effect but also synergistically contributed by the Fe^{3+} release, as the degradation of the MIL-88B was found in the SEM images when Gram-positive bacteria growing with liquid culture media, as shown in **Figure S10**. The degradation of MIL-88B could release large amounts of Fe^{3+}, and these released Fe^{3+} could thereby participate in Fenton-like reactions and result in antibacterial effects.[54,55] Therefore, we observed high bactericidal efficiency of MoU (99% for *S. aureus*) and M+U surfaces for Gram-positive bacteria. Notably, mechano-bactericidal effects of the MoU surfaces towards Gram-positive bacteria were still observed in the SEM images (main text Figure 4). However, distinguishing the contribution of the mechano-bactericidal part and the Fenton-like reaction part will require further study, and *E. coli* was used as the model for the quantitative study of MB actions of the MOF surfaces in this work.

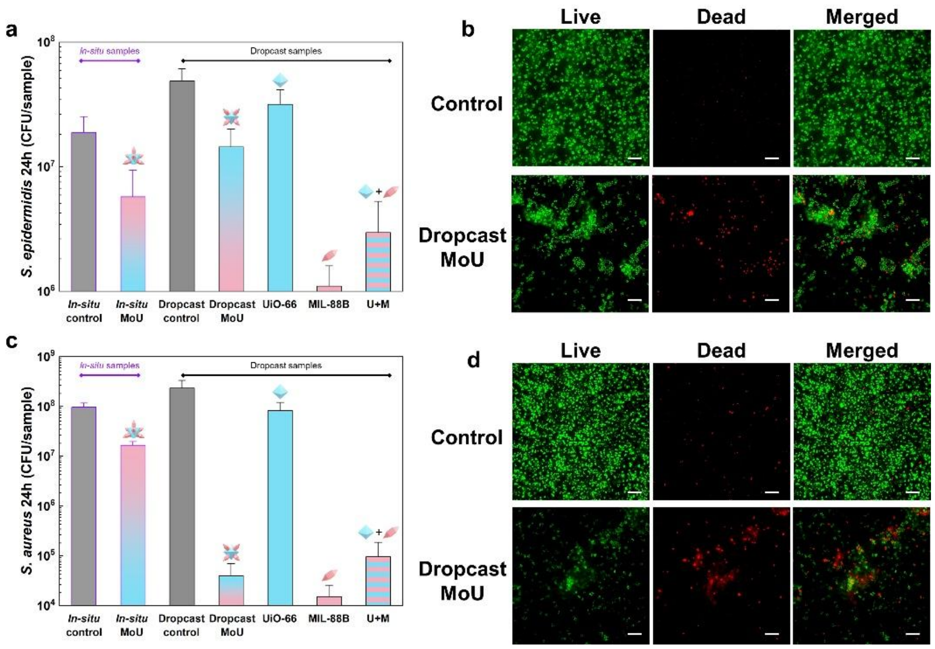

Figure S9 CFU results of attached bacteria after 24h growth: (a) *S. epidermidis* on *in-situ* MoU samples and dropcasting samples, including MoU, UiO-66, MIL-88B, and UiO-66 + MIL-88B (U+M); (c) *S. aureus* on *in-situ* MoU samples and dropcasting samples, including MoU, UiO-66, MIL-88B, and U+M, Data represent the mean ± standard deviation of three biological replicates. The live/dead fluorescent staining images of attached bacteria with 24h growth: (b) *S. epidermidis* on control and dropcast MoU surfaces; (d) *S. aureus* on control and dropcast MoU surfaces, green indicating live bacteria and red indicating dead bacteria, scale bar: 10 μm

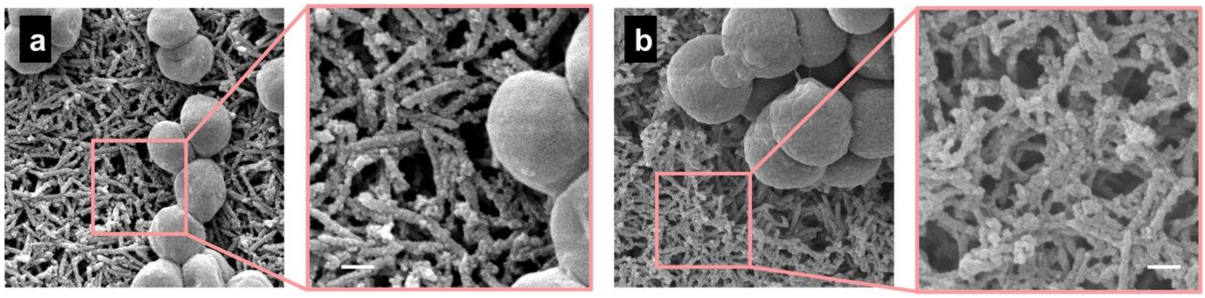

Figure S10 SEM images of dropcast MIL-88B surfaces after Gram-positive bacteria growth for 24h: (a) *S. epidermidis*, (b) *S. aureus*; degradation of MIL-88B was observed, scale bar: 200 nm

Section 11: Representative plate photographs of CFU counting

The representative CFU plate photographs of *E. coli*, *S. epidermidis*, and *S. aureus* after 24h growth on dropcast and *in-situ* MOF surfaces are presented in **Figure S11**. These raw data were used for constructing the CFU bar chart in the main text (**Figure 3a**). Dropcast MOF surfaces with a larger area in substrates (round glass slice, diameter 10 mm) presented higher colony numbers in control groups comparing with the *in-situ* surfaces with silicon chips as substrates (square, 6 mm * 6mm).

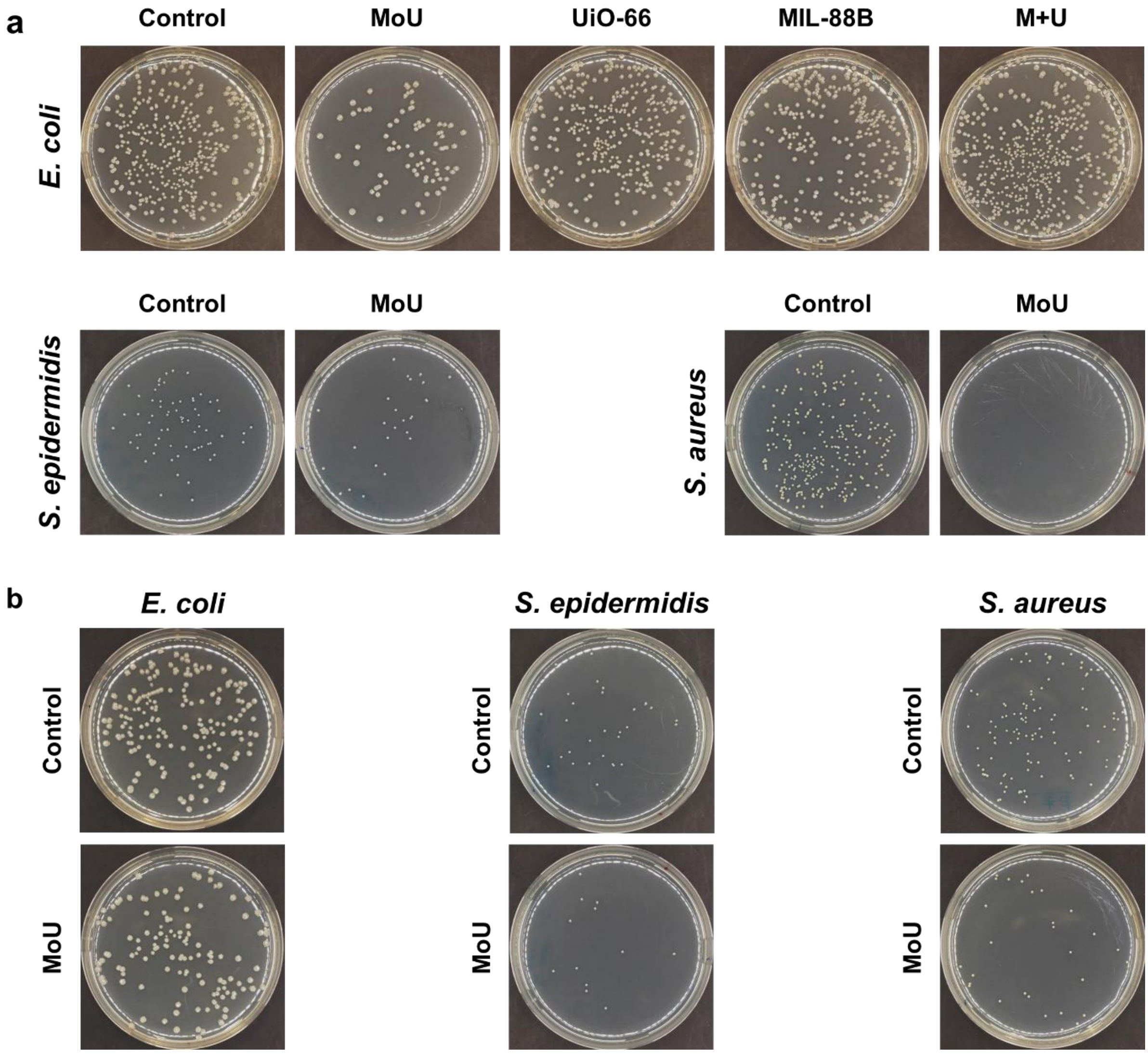

Figure S11 Representative CFU plate photographs of *E. coli, S. epidermidis,* and *S. aureus* after 24h growth on (a) dropcast and (b) *in-situ* MOF surfaces

Section 12: Effective bactericidal area analysis

The effective bactericidal area was analyzed by the previously reported methods.[56,57] SEM images of *in-situ*/dropcast MoU surfaces with three different regions were used for the uncovered area analysis. Dropcast MoU surfaces possess more covered areas (85%) than the *in-situ* MoU surfaces (64%), with detailed information in **Table S8**. Furthermore, due to the random four-pin up orientation of the nanopillars in dropcast MoU, more MIL-88B nanopillars of the MoU could also induce mechanical stress on bacteria, which could contribute to an elevated bactericidal efficiency in dropcast MoU surfaces compared to the *in-situ* MoU surfaces.

Table S8 The percentage of uncovered areas on *in-situ* and dropcast MoU surfaces

No.	*In-situ*	Dropcast
1	33%	14%
2	39%	20%
3	37%	12%
Mean	36%	15%
Stand. Div.	2.49%	3.40%

Section 13: Bactericidal efficiency of dropcast MoU surface for 72h

To investigate the long-term bactericidal performance of the MOF surfaces, plate counting of colony-forming unit (CFU) method was used to evaluate the attached bacteria on the dropcast MoU surfaces after 72h growth of *E. coli*. The LB culture medium was changed every 24 hours to provide fresh nutrients. As shown in **Figure S12**, the MoU surfaces demonstrated relatively higher bactericidal efficiency (51%) than UiO-66 (17%), MIL-88B (31%), and UiO-66 + MIL-88B (U+M) (9%). However, the bactericidal efficiency dropped from 83% (24h growth) to 51% (72h growth), which could be attributed to the coverage of the nanostructures by the debris. The debris including the dead bacteria could impede the contact of the planktonic bacteria to the nanostructures of the MB surfaces, leading to diminished bactericidal performance for certain MB surfaces.[58,59] Thus, a strategy to efficiently clean the MB surfaces would be required to provide long-term protection for our MOF MB surfaces.[60]

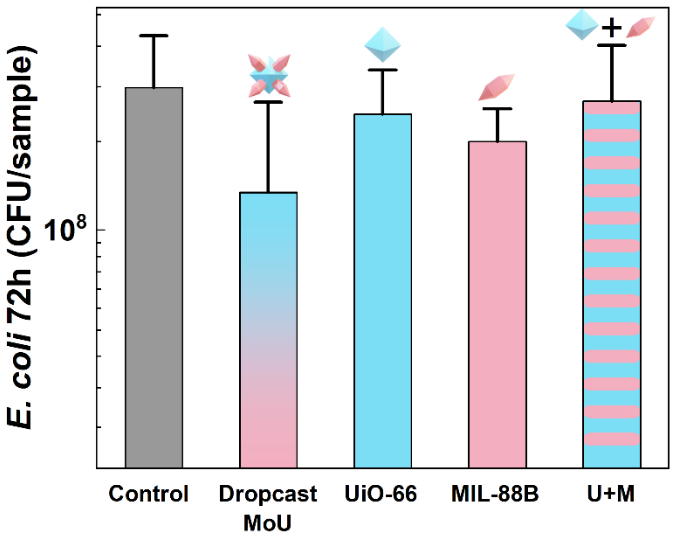

Figure S12 CFU results of attached *E. coli* with 72h growth on dropcasting samples, including MoU, UiO-66, MIL-88B, and UiO-66 + MIL-88B (U+M). Data represent the mean ± standard deviation of three biological replicates

Section 14: Tilted SEM images of *in-situ* MOF MB surfaces

To achieve better observation of the *in-situ* MOF MB surfaces as well as the interaction between the MOF structures and bacteria, tilted (45°) SEM images were acquired as shown in **Figure S13.** Tilted SEM images of the *in-situ* UiO-66 surfaces confirmed UiO-66 seamlessly grew on the substrate and provided a near horizontal triangle surface for the following MoU epitaxial growth. Tilted SEM images of *in-situ* MoU surfaces confirmed the one-pin up orientation of the MoU hybrid. Furthermore, with close-up views of *E. coli* and *S. aureus* along with the non-tilted SEM images in the main text and SI section 15, the MB actions were verified, as the *stretching*, *impaling,* and *mechanical injury* were observed with a lateral view with torn, deformed, and deflated bacterial envelopes, as highlighted in the yellow dashed circles.

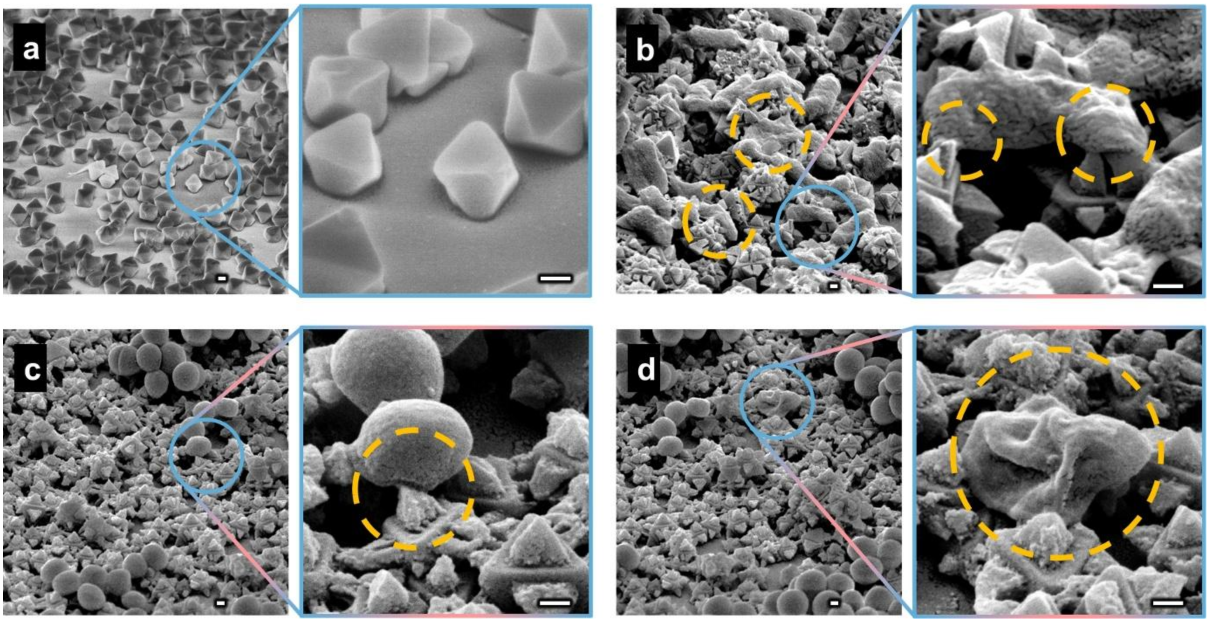

Figure S13 Tilted (45°) SEM images of (a) *in-situ* UiO-66 surfaces, *in-situ* MoU surfaces with attached (b) *E. coli*, (c, d) *S. aureus* after 24h growth; mechano-bactericidal actions were observed and highlighted in the yellow dashed circles. Scale bar 200 nm

Section 15: SEM images of attached bacteria on MOF surfaces

SEM is a great tool to reveal the interaction between bacteria and MOF surfaces. MB actions were found not only for *E. coli* and *S. epidermidis* but also for MDR *S. aureus* in both *in-situ* MoU and dropcast MoU (**Figure S14**), where direct impaling and mechanical injury were observed. However, the MB actions were rarely found on the sole UiO-66 and MIL-88B surfaces due to missing critical geometry features of MB surfaces, such as sharp tips and vertical orientation, as shown in **Figure S15**. Degradation of the MIL-88B was found in the Gram-positive bacteria samples as described in Section 10.

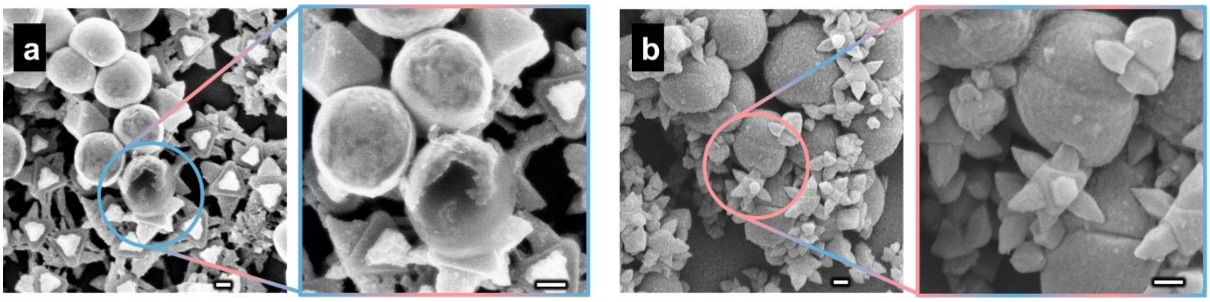

Figure S14 SEM images of attached bacteria on different MOF surfaces after 24h growth. *S. aureus* on (a) *in-situ* and (b) dropcast MoU surfaces, direct impaling and mechanical injury observed on *S. aureus*. Scale bar: 200 nm

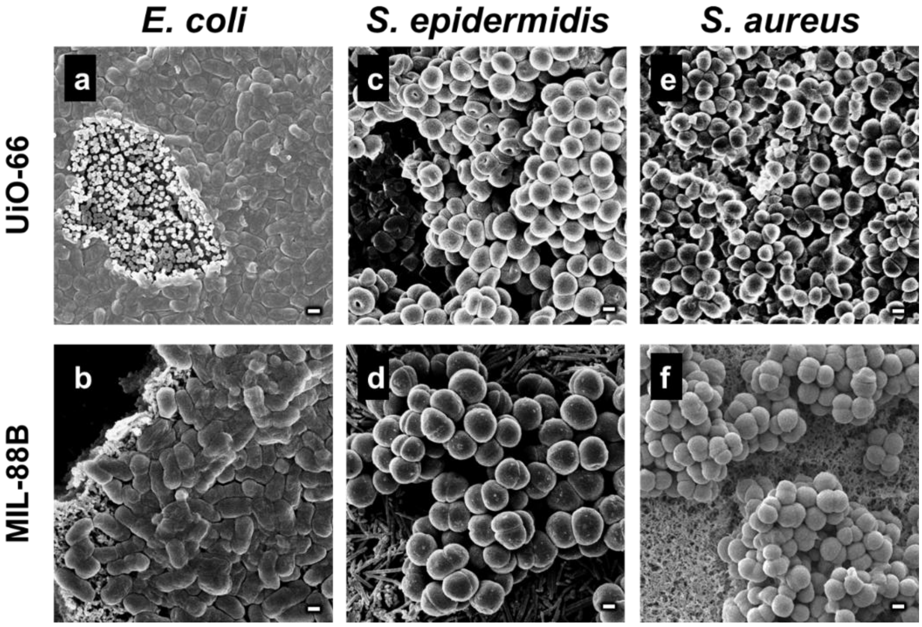

Figure S15 SEM images of attached bacteria on dropcast UiO-66 and MIL-88B with: *E. coli* (a) and (b); *S. epidermidis* (c) and (d); *S. aureus* (e) and (f). Mechano-bactericidal actions were rarely observed on dropcast UiO-66 or MIL-88B surfaces. Scale bar: 500 nm

Section 16: Stability of the MoU hybrid and MoU surfaces

Good stability of MoU hybrid and MoU surfaces is required to provide reliable protection from bacterial attachment. For the MoU hybrid, the seamless interfaces have been verified in previously reported epitaxial MOF growth work,[61] confirming a stable connection between the UiO-66 cores and MIL-88B satellites. Our synthesized MoU hybrid presented excellent structural stability, revealed by the preserved intact structure after ultrasonication treatment and immersion in water, culture media, and ethanol, as shown in **Figure S16a**. We also verified the stability of the MoU surfaces by SEM images after different treatments. Ultrasonication was applied to remove the dangling MOFs and debris after the synthesis of *in-situ* MoU surfaces. MoU hybrids maintained full coverage on the silicon surface after sonification and bio-tests, indicating strong adhesion between the MOFs and substrates (**Figure S16b**). For the *ex-situ* dropcast MoU surface, the same fixation protocol (80 °C, 3h) was applied according to our previous work.[12,57,62] The obtained *ex-situ* MoU dropcast surfaces maintained full coverage over the glass slice and were not broken nor washed away after bactericidal evaluation and series dehydration (40%, 50%, 60%, 70%, 80%, 90%, and 100% ethanol), where several times of pipetting were involved (**Figure S16c**). The intact structure of the MoU hybrids and MoU surfaces confirmed good stability and adhesion required for the MB action study in this work. However, for applications require strong adhesion, adhesive layers, such as polydopamine, hydrogel, could be introduced as reported in many works.[63,64]

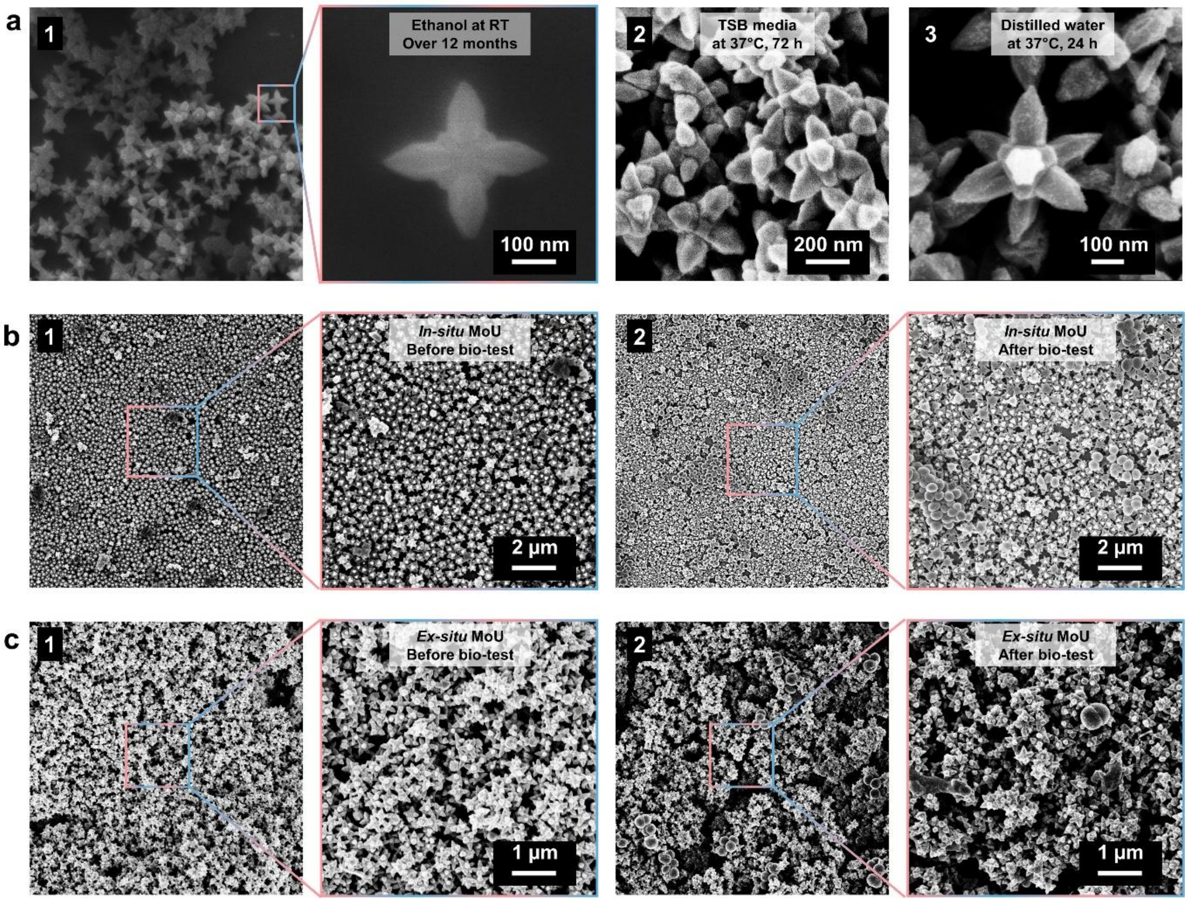

Figure S16 SEM images of MoU hybrids and surfaces. (a) MoU hybrids maintained their intact structures after immersion in solvents under different conditions: (1) ethanol at room temperature (RT, around 20 °C) for 12 months, (2) TSB media at 37 °C for 72 h, (3) distilled water at 37 °C for 24 h. (b) *In-situ* MoU surfaces maintained full surface coverage: (1) before bio-tests, (2) after bio-tests (24h, 37 °C, with *S. epidermidis*) with several times of pipetting. (c) (b) *Ex-situ* dropcast MoU surfaces maintained full surface coverage: (1) before bio-tests, (2) after bio-tests (72h, 37 °C, with *S. epidermidis*) with several times of pipetting

Section 17: Stress analysis simulation

The *in-situ* MoU surfaces were used for stress analysis as the geometry features can be obtained through SEM and TEM images. The model of the *in-situ* MoU surface was first simplified as shown in **Figure S17ab**. *E.coli* and *S. aureus* were selected as the bacteria models as they were tested in this work and with many reported parameters, such as adhesion force.[65–67] The simulation was performed using the software Ansys 2024 R1 as the environment of the finite element analysis. The size information and other parameters used in the simulation are summarized in **Table S9**. Notably, the chemical interactions between the MOF particles and the bacterial lipids were not taken into consideration in this simulation. However, the MOF surfaces have been proven to be hydrophobic in Section 6, and the hydrophobicity of the nanostructures has been reported to be beneficial for penetration into bacteria.[57,62]

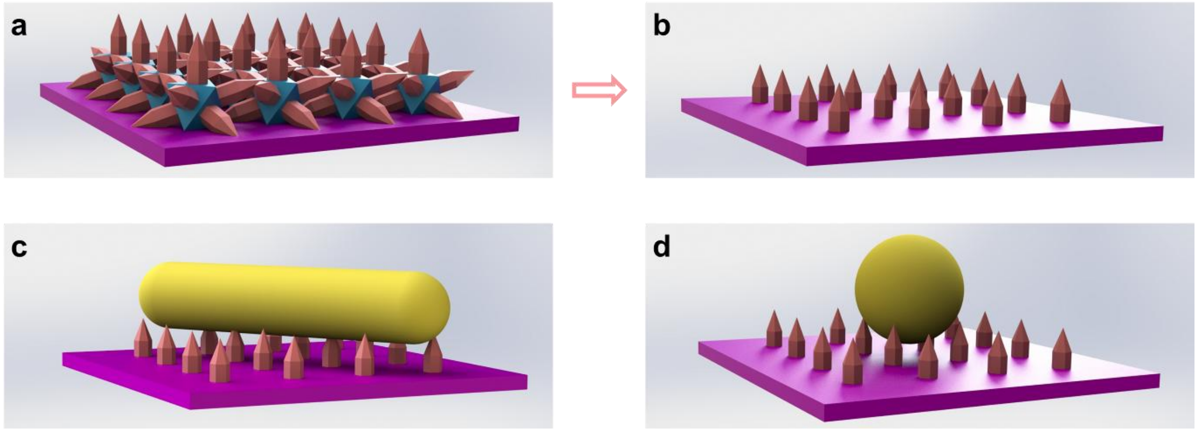

Figure S17 Structure modeling of the (a) *in-situ* MoU surface, (b) simplified *in-situ* MoU surface; (c) *E. coli* and (d) *S. aureus* on simplified *in-situ* MoU surface

Table S9 Parameters for numerical computation of stress distribution for bacteria on MOF MB surfaces

**Object**	**Parameters**
E. coli	Cylinder diameter: 1 µm
	Cylinder length: 2 µm
	Hemisphere diameter: 500 nm
	Density: 1105 kg m^{-3}[68]
	Young's modulus: 5 MPa[69]
	Adhesion force: 10 nN[70]
S. aureus	Sphere diameter: 800 nm
	Density: 1105 kg m^{-3}
	Young's modulus: 1.5 MPa[69]
	Adhesion force: 10 nN[67]
**MOF surface (MIL-88B nanopillar)**	Side length of hexagon: 97 nm
	Height of the Hexagonal prism: 131 nm
	Length of the tip:172 nm
	Pitch distance: 527 nm
	Young's modulus: 7.7 GPa[71]

Section 18: Fabrication of MOF MB surfaces

All the starting chemicals, including $ZrCl_4$, $Fe(NO_3)_3\cdot 9H_2O$, Benzene-1,4-dicarboxylic acid (BDC), acetic acid (AcOH), acetonitrile (CH_3CN), and *N,N*-Dimethylformamide (DMF) were obtained commercially (Sigma Aldrich) and used as received without further purification.

UiO-66, MIL-88B, and MIL-88B on UiO-66 (MoU) were obtained by modifying previous work, as listed in detail below.

UiO-66(Zr): UiO-66 was synthesized based on a reported method.[72] $ZrCl_4$ solution (DMF, 36 mM) and BDC solution (DMF, 36 mM) were mixed, and then AcOH was added to the solution for a 10 min stirring mixing at room temperature. The molar ratio of $ZrCl_4$: BDC : DMF: AcOH is 1:1:720:580. The obtained mixture was then transferred to a Teflon-lined autoclave. Silicon chips (6 mm * 6mm) were loaded to the bottom of the autoclave to obtain the *in-situ* UiO-66 surfaces[45]. The autoclave was held at 120 °C for 24h. After cooling to room temperature, the obtained powder was filtered and subsequently washed by DMF 3 times and ethanol 3 times through redispersion. The *in-situ* UiO-66 samples were washed by DMF 3 times and ethanol 3 times through pipette rinsing, followed by sonification at 80% power to remove the dangling particles. Obtained UiO-66 powder and *in-situ* UiO-66 were dried in a static vacuum oven at 60 °C overnight.

MIL-88B(Fe): MIL-88B was synthesized based on a reported method.[73] $Fe(NO_3)_3\cdot 9H_2O$ solution (DMF, 200 mM) and BDC solution (DMF, 100 mM) were mixed, and then CH_3CN was added to the solution for a 10 min stirring mixing at room temperature. The molar ratio of $Fe(NO_3)_3\cdot 9H_2O$: BDC : DMF: CH_3CN is 1:1:200:290. The obtained mixture was then transferred to Pyrex tubes and held at 90 °C for 5h. After cooling to room temperature, the obtained powder was filtered and subsequently washed by DMF 3 times and ethanol 3 times through redispersion. Obtained MIL-88B powder was dried in a static vacuum oven at 60 °C overnight.

MIL-88B on UiO-66 (MoU): MoU was synthesized based on reported methods.[61,73] $Fe(NO_3)_3\cdot 9H_2O$ solution (DMF, 200 mM, 3 mL) and BDC solution (DMF, 100 mM, 6mL) were mixed, and then CH_3CN (9 mL) was added to the solution for a 10 min stirring mixing at room temperature. Thereafter, 43 mg UiO-66 (in 2 mL DMF) was added to the mixture, followed by 5 min sonification. The obtained mixture was then transferred to Pyrex tubes where an *in-situ* UiO-66 chip was loaded to the bottom of the tubes and then held at 90 °C for 5h. After cooling to room temperature, the obtained powder was filtered and subsequently washed by DMF 3 times and ethanol 3 times through redispersion. The *in-situ* MoU samples were washed by DMF 3 times and ethanol 3 times through pipette rinsing, followed by sonification at 80% power to remove the dangling particles. Obtained MoU powder and in-situ MoU were dried in a static vacuum oven at 60 °C overnight.

Dropcast MOF MB surfaces: MoU, UiO-66, and MIL-88B were dispersed in ethanol at a concentration of 5 mg mL^{-1}. UiO-66 and MIL-88B solution were mixed at the volume ratio of 1:1, denoted as MIL-88B plus UiO-66 (M+U). After sonification, 50 μL MOF solution was dropcast on the round glass slide (diameter 1 cm). After drying at room temperature, the samples were loaded in an 80 °C oven for 3 h to fix the MOFs to the surfaces. All the samples were dried in a static vacuum oven at 60 °C overnight before tests.

Reference for supporting information

[1] E. P. Ivanova, J. Hasan, H. K. Webb, V. K. Truong, G. S. Watson, J. A. Watson, V. A. Baulin, S. Pogodin, J. Y. Wang, M. J. Tobin, C. Löbbe, R. J. Crawford, *Small* **2012**, *8*, 2489.
[2] E. P. Ivanova, J. Hasan, H. K. Webb, G. Gervinskas, S. Juodkazis, V. K. Truong, A. H. F. Wu, R. N. Lamb, V. A. Baulin, G. S. Watson, J. A. Watson, D. E. Mainwaring, R. J. Crawford, *Nat. Commun.* **2013**, *4*, 2838.
[3] G. S. Watson, D. W. Green, L. Schwarzkopf, X. Li, B. W. Cribb, S. Myhra, J. A. Watson, *Acta Biomater.* **2015**, *21*, 109.
[4] X. Li, G. S. Cheung, G. S. Watson, J. A. Watson, S. Lin, L. Schwarzkopf, D. W. Green, *Nanoscale* **2016**, *8*, 18860.
[5] S. M. Kelleher, O. Habimana, J. Lawler, B. O' Reilly, S. Daniels, E. Casey, A. Cowley, *ACS Appl. Mater. Interfaces* **2016**, *8*, 14966.
[6] K. Nowlin, A. Boseman, A. Covell, D. LaJeunesse, *J. R. Soc. Interface* **2015**, *12*, 20140999.
[7] Y. Chen, J. Gao, J. Ao, J. Zhang, R. Jiang, Z. Zhang, Z. Liu, J. Zhao, L. Ren, *Colloids Surf. B Biointerfaces* **2023**, *224*, 113229.
[8] D. P. Linklater, H. K. D. Nguyen, C. M. Bhadra, S. Juodkazis, E. P. Ivanova, *Nanotechnology* **2017**, *28*, 245301.
[9] E. Vassallo, M. Pedroni, T. Silvetti, S. Morandi, S. Toffolatti, G. Angella, M. Brasca, *Mater. Sci. Eng. C* **2017**, *80*, 117.
[10] J. Hasan, S. Raj, L. Yadav, K. Chatterjee, *RSC Adv.* **2015**, *5*, 44953.
[11] V. T. H. Pham, V. K. Truong, A. Orlowska, S. Ghanaati, M. Barbeck, P. Booms, A. J. Fulcher, C. M. Bhadra, R. Buividas, V. Baulin, C. J. Kirkpatrick, P. Doran, D. E. Mainwaring, S. Juodkazis, R. J. Crawford, E. P. Ivanova, *ACS Appl. Mater. Interfaces* **2016**, *8*, 22025.
[12] S. Pandit, Z. Cao, V. R. S. S. Mokkapati, E. Celauro, A. Yurgens, M. Lovmar, F. Westerlund, J. Sun, I. Mijakovic, *Adv. Mater. Interfaces* **2018**, *5*, 1701331.
[13] D. P. Linklater, M. De Volder, V. A. Baulin, M. Werner, S. Jessl, M. Golozar, L. Maggini, S. Rubanov, E. Hanssen, S. Juodkazis, E. P. Ivanova, *ACS Nano* **2018**, *12*, 6657.
[14] T. Diu, N. Faruqui, T. Sjöström, B. Lamarre, H. F. Jenkinson, B. Su, M. G. Ryadnov, *Sci. Rep.* **2014**, *4*, 7122.
[15] C. M. Bhadra, V. Khanh Truong, V. T. H. Pham, M. Al Kobaisi, G. Seniutinas, J. Y. Wang, S. Juodkazis, R. J. Crawford, E. P. Ivanova, *Sci. Rep.* **2015**, *5*, 16817.
[16] J. Hasan, S. Jain, K. Chatterjee, *Sci. Rep.* **2017**, *7*, 41118.
[17] Y. Cao, B. Su, S. Chinnaraj, S. Jana, L. Bowen, S. Charlton, P. Duan, N. S. Jakubovics, J. Chen, *Sci. Rep.* **2018**, *8*, 1071.
[18] F. Hizal, I. Zhuk, S. Sukhishvili, H. J. Busscher, H. C. van der Mei, C.-H. Choi, *ACS Appl. Mater. Interfaces* **2015**, *7*, 20304.
[19] S. Wu, F. Zuber, J. Brugger, K. Maniura-Weber, Q. Ren, *Nanoscale* **2016**, *8*, 2620.
[20] S. Wu, F. Zuber, K. Maniura-Weber, J. Brugger, Q. Ren, *J. Nanobiotechnology* **2018**, *16*, 20.
[21] M. N. Dickson, E. I. Liang, L. A. Rodriguez, N. Vollereaux, A. F. Yee, *Biointerphases* **2015**, *10*, 021010.
[22] X. Lu, J. Ye, D. Zhang, R. Xie, R. F. Bogale, Y. Sun, L. Zhao, Q. Zhao, G. Ning, *J. Inorg. Biochem.* **2014**, *138*, 114.
[23] M. Berchel, T. L. Gall, C. Denis, S. L. Hir, F. Quentel, C. Elléouet, T. Montier, J.-M. Rueff, J.-Y. Salaün, J.-P. Haelters, G. B. Hix, P. Lehn, P.-A. Jaffrès, *New J. Chem.* **2011**, *35*, 1000.
[24] Y. Liu, X. Xu, Q. Xia, G. Yuan, Q. He, Y. Cui, *Chem. Commun.* **2010**, *46*, 2608.
[25] S. W. Jaros, M. F. C. Guedes da Silva, M. Florek, M. C. Oliveira, P. Smoleński, A. J. L. Pombeiro, A. M. Kirillov, *Cryst. Growth Des.* **2014**, *14*, 5408.
[26] X. Wang, D. Zhao, A. Tian, J. Ying, *Dalton Trans.* **2014**, *43*, 5211.
[27] A. R. Abbasi, K. Akhbari, A. Morsali, *Ultrason. Sonochem.* **2012**, *19*, 846.
[28] H. S. Rodríguez, J. P. Hinestroza, C. Ochoa-Puentes, C. A. Sierra, C. Y. Soto, *J. Appl. Polym. Sci.* **2014**, *131*, DOI 10.1002/app.40815.
[29] C. Chiericatti, J. C. Basilico, M. L. Zapata Basilico, J. M. Zamaro, *Microporous Mesoporous Mater.* **2012**, *162*, 60.

[30] M. P. Arpa Sancet, M. Hanke, Z. Wang, S. Bauer, C. Azucena, H. K. Arslan, M. Heinle, H. Gliemann, C. Wöll, A. Rosenhahn, *Biointerphases* **2013**, *8*, 29.
[31] S. M. Sheta, S. M. El-Sheikh, M. M. Abd-Elzaher, *Dalton Trans.* **2018**, *47*, 4847.
[32] Z. Yu, X. Li, Z. Wang, Y. Fan, W. Zhao, D. Li, D. Xu, T. Gu, F. Wang, *Adv. Mater.* **2024**, 2407409.
[33] V. Pezeshkpour, S. A. Khosravani, M. Ghaedi, K. Dashtian, F. Zare, A. Sharifi, R. Jannesar, M. Zoladl, *Ultrason. Sonochem.* **2018**, *40*, 1031.
[34] I. R. Colinas, M. D. Rojas-Andrade, I. Chakraborty, S. R. J. Oliver, *CrystEngComm* **2018**, *20*, 3353.
[35] P. K. Kermanshahi, K. Akhbari, *RSC Adv.* **2024**, *14*, 5601.
[36] C. Tamames-Tabar, E. Imbuluzqueta, N. Guillou, C. Serre, S. R. Miller, E. Elkaïm, P. Horcajada, M. J. Blanco-Prieto, *CrystEngComm* **2014**, *17*, 456.
[37] W. Zhuang, D. Yuan, J.-R. Li, Z. Luo, H.-C. Zhou, S. Bashir, J. Liu, *Adv. Healthc. Mater.* **2012**, *1*, 225.
[38] S. Aguado J. Quirós, J. Canivet, D. Farrusseng, K. Boltes, R. Rosal, *Chemosphere* **2014**, *113*, 188.
[39] Y. Yuan, Y. Zhang, *Nanomedicine Nanotechnol. Biol. Med.* **2017**, *13*, 2199.
[40] Y. Cheng, X. Ma, T. Franklin, R. Yang, C. I. Moraru, *Annu. Rev. Food Sci. Technol.* **2023**, *14*, 449.
[41] D. P. Linklater, V. A. Baulin, S. Juodkazis, R. J. Crawford, P. Stoodley, E. P. Ivanova, *Nat. Rev. Microbiol.* **2021**, *19*, 8.
[42] R. S. Forgan, *Chem. Sci.* **2020**, *11*, 4546.
[43] B. Shan, J. B. James, M. R. Armstrong, E. C. Close, P. A. Letham, K. Nikkhah, Y. S. Lin, B. Mu, *J. Phys. Chem. C* **2018**, *122*, 2200.
[44] K. M. D. Sisican, K. A. S. Usman, C. J. O. Bacal, Y. D. G. Edañol, M. T. Conato, *Cryst. Growth Des.* **2023**, *23*, 8509.
[45] M. Miyamoto, S. Kohmura, H. Iwatsuka, Y. Oumi, S. Uemiya, *CrystEngComm* **2015**, *17*, 3422.
[46] H. Sobhi, M. E. Matthews, B. Grandy, J. Masnovi, A. T. Riga, *J. Therm. Anal. Calorim.* **2008**, *93*, 535.
[47] M. Saini, Y. Singh, P. Arora, V. Arora, K. Jain, *World J. Clin. Cases* **2015**, *3*, 52.
[48] S.-N. Kim, Y.-R. Lee, S.-H. Hong, M.-S. Jang, W.-S. Ahn, *Catal. Today* **2015**, *245*, 54.
[49] Y. Wu, Y. Fang, J. Fu, L. He, D. M. Kabtamu, L. Matović, F. Li, J. Li, *J. Environ. Chem. Eng.* **2022**, *10*, 108556.
[50] J. E. Ellis, S. E. Crawford, K.-J. Kim, *Mater. Adv.* **2021**, *2*, 6169.
[51] M. Fu, B. Chai, J. Yan, C. Wang, G. Fan, G. Song, F. Xu, *Appl. Phys. A* **2021**, *127*, 928.
[52] T. Yamashita, P. Hayes, *Appl. Surf. Sci.* **2008**, *254*, 2441.
[53] Z. Li, R. Hu, S. Ye, J. Song, L. Liu, J. Qu, *J. Mater. Sci.* **2021**, *56*, 1577.
[54] L. Bondarenko, R. Baimuratova, A. Dzeranov, D. Pankratov, A. Kicheeva, E. Sushko, N. Kudryasheva, R. Valeev, N. Tropskaya, G. Dzhardimalieva, K. Kydralieva, *New J. Chem.* **2024**, *48*, 10142.
[55] R. Zhu, M. Cai, T. Fu, D. Yin, H. Peng, S. Liao, Y. Du, J. Kong, J. Ni, X. Yin, *Pharmaceutics* **2023**, *15*, 1599.
[56] S. Rahimi, T. Lovmar, A. Aulova, S. Pandit, M. Lovmar, S. Forsberg, M. Svensson, R. Kádár, I. Mijakovic, *Nanomaterials* **2023**, *13*, 1605.
[57] S. Pandit, K. Gaska, V. R. S. S. Mokkapati, E. Celauro, A. Derouiche, S. Forsberg, M. Svensson, R. Kádár, I. Mijakovic, *Small* **2020**, *16*, 1904756.
[58] Z. Liu, Y. Yi, L. Song, Y. Chen, L. Tian, J. Zhao, L. Ren, *Acta Biomater.* **2022**, *141*, 198.
[59] R. Jiang, Y. Yi, L. Hao, Y. Chen, L. Tian, H. Dou, J. Zhao, W. Ming, L. Ren, *ACS Appl. Mater. Interfaces* **2021**, *13*, 60865.
[60] H.-K. Kim, H. W. Baek, H.-H. Park, Y.-S. Cho, *Colloids Surf. B Biointerfaces* **2024**, *234*, 113729.
[61] O. Kwon, J. Y. Kim, S. Park, J. H. Lee, J. Ha, H. Park, H. R. Moon, J. Kim, *Nat. Commun.* **2019**, *10*, 3620.
[62] V. Ghai, S. Pandit, M. Svensso, R. Larsson, A. Matic, R. Ngaloy, S. P. Dash, A. Terry, K. Nygård, I. Mijakovic, R. Kádár, *Adv. Funct. Mater.* **2024**, 2406875.

[63] X. Wang, J. Hou, F. Chen, X. Meng, *Sep. Purif. Technol.* **2020**, *236*, 116239.
[64] Z. Yu, X. Li, X. Li, B. Zheng, D. Li, D. Xu, F. Wang, *Adv. Funct. Mater.* **2023**, *33*, 2305995.
[65] A. Harimawan, A. Rajasekar, Y.-P. Ting, *J. Colloid Interface Sci.* **2011**, *364*, 213.
[66] A. Elbourne, J. Chapman, A. Gelmi, D. Cozzolino, R. J. Crawford, V. K. Truong, *J. Colloid Interface Sci.* **2019**, *546*, 192.
[67] F. Alam, K. Balani, *J. Mech. Behav. Biomed. Mater.* **2017**, *65*, 872.
[68] I. Lee, J. Kim, R. Kwak, J. Lee, *Adv. Funct. Mater.* **2023**, *33*, 2213650.
[69] T. Liu, Q. Cui, Q. Wu, X. Li, K. Song, D. Ge, S. Guan, *J. Phys. Chem. B* **2019**, *123*, 8686.
[70] E. P. Ivanova, D. P. Linklater, M. Werner, V. A. Baulin, X. Xu, N. Vrancken, S. Rubanov, E. Hanssen, J. Wandiyanto, V. K. Truong, A. Elbourne, S. Maclaughlin, S. Juodkazis, R. J. Crawford, *Proc. Natl. Acad. Sci.* **2020**, *117*, 12598.
[71] F. A. Son, K. M. Fahy, M. A. Gaidimas, C. S. Smoljan, M. C. Wasson, O. K. Farha, *Commun. Chem.* **2023**, *6*, 1.
[72] J. H. Cavka, S. Jakobsen, U. Olsbye, N. Guillou, C. Lamberti, S. Bordiga, K. P. Lillerud, *J. Am. Chem. Soc.* **2008**, *130*, 13850.
[73] X.-G. Wang, L. Xu, M.-J. Li, X.-Z. Zhang, *Angew. Chem. Int. Ed.* **2020**, *59*, 18078.
